# Differentiated function and localisation of SPO11-1 and PRD3 on the chromosome axis during meiotic DSB formation in *Arabidopsis thaliana*

**DOI:** 10.1371/journal.pgen.1010298

**Published:** 2022-07-20

**Authors:** Christophe Lambing, Pallas Kuo, Jaeil Kim, Kim Osman, Amy Leanne Whitbread, Jianhua Yang, Kyuha Choi, F. Chris H. Franklin, Ian R. Henderson

**Affiliations:** 1 Department of Plant Sciences, University of Cambridge, Cambridge, United Kingdom; 2 Rothamsted Research, Harpenden, United Kingdom; 3 School of Biosciences, University of Birmingham, Edgbaston, Birmingham, United Kingdom; 4 Department of Life Sciences, Pohang University of Science and Technology, Pohang, Republic of Korea; Fudan University, CHINA

## Abstract

During meiosis, DNA double-strand breaks (DSBs) occur throughout the genome, a subset of which are repaired to form reciprocal crossovers between chromosomes. Crossovers are essential to ensure balanced chromosome segregation and to create new combinations of genetic variation. Meiotic DSBs are formed by a topoisomerase-VI-like complex, containing catalytic (e.g. SPO11) proteins and auxiliary (e.g. PRD3) proteins. Meiotic DSBs are formed in chromatin loops tethered to a linear chromosome axis, but the interrelationship between DSB-promoting factors and the axis is not fully understood. Here, we study the localisation of SPO11-1 and PRD3 during meiosis, and investigate their respective functions in relation to the chromosome axis. Using immunocytogenetics, we observed that the localisation of SPO11-1 overlaps relatively weakly with the chromosome axis and RAD51, a marker of meiotic DSBs, and that SPO11-1 recruitment to chromatin is genetically independent of the axis. In contrast, PRD3 localisation correlates more strongly with RAD51 and the chromosome axis. This indicates that PRD3 likely forms a functional link between SPO11-1 and the chromosome axis to promote meiotic DSB formation. We also uncovered a new function of SPO11-1 in the nucleation of the synaptonemal complex protein ZYP1. We demonstrate that chromosome co-alignment associated with ZYP1 deposition can occur in the absence of DSBs, and is dependent on SPO11-1, but not PRD3. Lastly, we show that the progression of meiosis is influenced by the presence of aberrant chromosomal connections, but not by the absence of DSBs or synapsis. Altogether, our study provides mechanistic insights into the control of meiotic DSB formation and reveals diverse functional interactions between SPO11-1, PRD3 and the chromosome axis.

## Author summary

Most eukaryotes rely on the formation of gametes with half the number of chromosomes for sexual reproduction. Meiosis is a specialised type of cell division essential for the transition between a diploid and a haploid stage during gametogenesis. In early meiosis, programmed-DNA double strand breaks (DSBs) occur across the genome. These DSBs are processed by a set of proteins and the broken ends are repaired using the genetic information from the homologous chromosomes. These reciprocal exchanges of information between two chromosomes are called crossovers. Crossovers physical link chromosomes in pairs which is essential to ensure their correct segregation during the two rounds of meiotic division. Crossovers are also essential for the creation of genetic diversity as they break genetic linkages to form novel allelic blocks. The formation of DSBs is not completely understood in plants. Here we studied the function of SPO11-1 and PRD3, two proteins involved in the formation of DSBs in Arabidopsis. We discovered functional differences in their respective mode of recruitment to the chromosomes, their interactions with proteins forming the chromosome core and their roles in chromosome co-alignment. These indicate that, although SPO11-1 and PRD3 share a role in the formation of DSBs, the two proteins have additional and distinct roles beside DSB formation.

## Introduction

Meiosis is a specialized cell division that is required for sexual reproduction and leads to the generation of genetic diversity [1]. Meiosis halves the chromosome number of the genome to form haploid gametes (e.g. sperm and egg cells), which upon fusion, restore the diploid state [1]. Furthermore, programmed DNA double-strand breaks (DSBs) are formed during meiosis and are repaired via homologous recombination between each pair of chromosomes to generate crossovers and non-crossovers [1]. Crossovers consist of reciprocal exchanges of genetic information between homologous chromosomes, which break pre-existing genetic linkages to form new combinations of alleles [1]. Meiotic DSB formation and repair are tightly regulated such that the integrity of the genome is preserved, while allowing creation of genetic diversity.

Meiotic DSBs are formed by a conserved topoisomerase-VI-like complex containing SPO11 and MTOPVIB [2,3]. In Arabidopsis, two SPO11 proteins are required for meiotic DSB formation; SPO11-1 and SPO11-2, in addition to the auxiliary proteins PRD1, PRD2, PRD3 and DFO [4–8]. During DSB formation, SPO11 remains covalently attached to the DNA 5′ ends at the break sites, allowing immunoprecipitation of SPO11-oligonucleotide complexes followed by sequencing, and thus identification of DSB locations [9–11]. Mapping SPO11-oligonucleotides genome-wide in plants, animals and fungi has revealed that meiotic DSBs are heterogeneously distributed throughout genomes [9–13]. For example, SPO11 DSB hotspots are associated with gene promoter regions that are nucleosome depleted in budding yeast [14]. Similarly, Arabidopsis SPO11-1 DSB hotspots are located in nucleosome-depleted regions at the 5′ and 3′ ends of genes and in specific transposons families [10]. In contrast, mice and human SPO11 DSB hotspots are spatially controlled by PRDM9 and are associated with intergenic regions and specific DNA motifs [15–17]. Epigenetic information on the chromatin and DNA also influences DSB formation [10,18]. For example, H3K9me2 and DNA methylation were found to repress DSB frequency in Arabidopsis, including within transposable elements [18–20].

Meiotic DSB formation occurs in the context of a linear chromosome axis, onto which the chromatin is organized in loop-axis arrays [21,22]. The Arabidopsis chromosome axis is composed of cohesin complexes containing REC8, the coiled-coil proteins ASY3 (a functional ortholog of Red1, SYCP2 and SYCP3) and ASY4, and the HORMA-containing protein ASY1 (a functional ortholog of Hop1, HORMAD1 and HORMAD2) [23–32]. REC8 cohesin organizes the chromatin around an axial structure, while ASY3 and ASY1 promote inter-homolog recombination [22,28,29,33–35]. The co-alignment of the two homologous chromosome axes leads to the formation of the synaptonemal complex, which consists of transverse filaments connecting the two axes and forming a tripartite structure that influences crossover formation [36–38]. In Arabidopsis and budding yeast, chromatin immunoprecipitation and sequencing (ChIP-seq) of REC8 revealed that cohesin is enriched in regions depleted of SPO11-oligonucleotides, consistent with DSBs forming in the chromatin loops away from the axis sites [33,34,39,40]. Depletion of meiotic cohesin causes a reduction in DSBs and early recombination markers in mice and plants [33,41,42]. A link between the axis and DSB formation was further demonstrated in budding yeast, where the PHD finger protein Spp1 simultaneously interacts with Mer2 on the chromosome axis, and H3K4me3 on the chromatin loops [43,44]. Through these interactions, Spp1 supports the tethering of recombination hotspots to the axis during DSB formation.

Many aspects of the spatial and temporal control of meiotic DSB formation remain elusive. For instance, mechanistic links between DSB formation and the chromosome axis are well documented in budding yeast [39,43–44], but less so in plants. In budding yeast and mice, numerical control of DSB formation is established by inter-homolog engagement, whereby connections between homologous chromosomes are sufficient to suppress formation of DSBs [45–47]. A second pathway limiting DSB numbers is dependent on Tel1 and Mec1 kinases in budding yeast [48]. In Arabidopsis, the homolog of Tel1 (ATM) is a negative regulator of meiotic DSB formation [49], but whether other pathways are present remains unclear.

To gain insights into meiotic DSB control in Arabidopsis, we used immunocytogenetic assays to explore the interrelationship of key components of the recombination machinery and chromosome axis. A recent ASY1 immunoprecipitation pull-down assay combined with mass spectrometry identified interacting proteins in Brassica [32]. Among the proteins co-immunoprecipitated, PRD3 was the only DSB-promoting factor identified [32]. This prompted us to focus our study on SPO11-1 and PRD3, and to investigate their respective localisation and function in relation to the chromosome axis. This revealed distinct localisation of PRD3 and SPO11-1 on meiotic chromosomes. Specifically, we found that PRD3 is relatively enriched on the chromosome axis, while SPO11-1 is enriched on the chromatin loops. We demonstrate that the association of PRD3 with the axis correlates with the timing of DSB formation during prophase I. We also show that the linear meiotic chromosome axis is required for the formation of DSBs, but not for the recruitment of SPO11-1, and that PRD3 physically interacts with several components of the chromosome axis. We identified a new function of SPO11-1 in the nucleation of the SC transverse filament protein ZYP1 [50], which is independent of DSB formation and not shared with PRD3. Lastly, we revealed that a delay in meiotic progression is linked with aberrant chromosomal connections, rather than the absence of recombination or synapsis. Our data provide mechanistic insights into the function of the DSB complex and the role of the chromosome axis in the initiation of meiotic recombination in plants.

## Results

### Dynamics of SPO11-1 foci are distinct from those of RAD51 and γH2AX during meiotic prophase I

**Fig 1:**
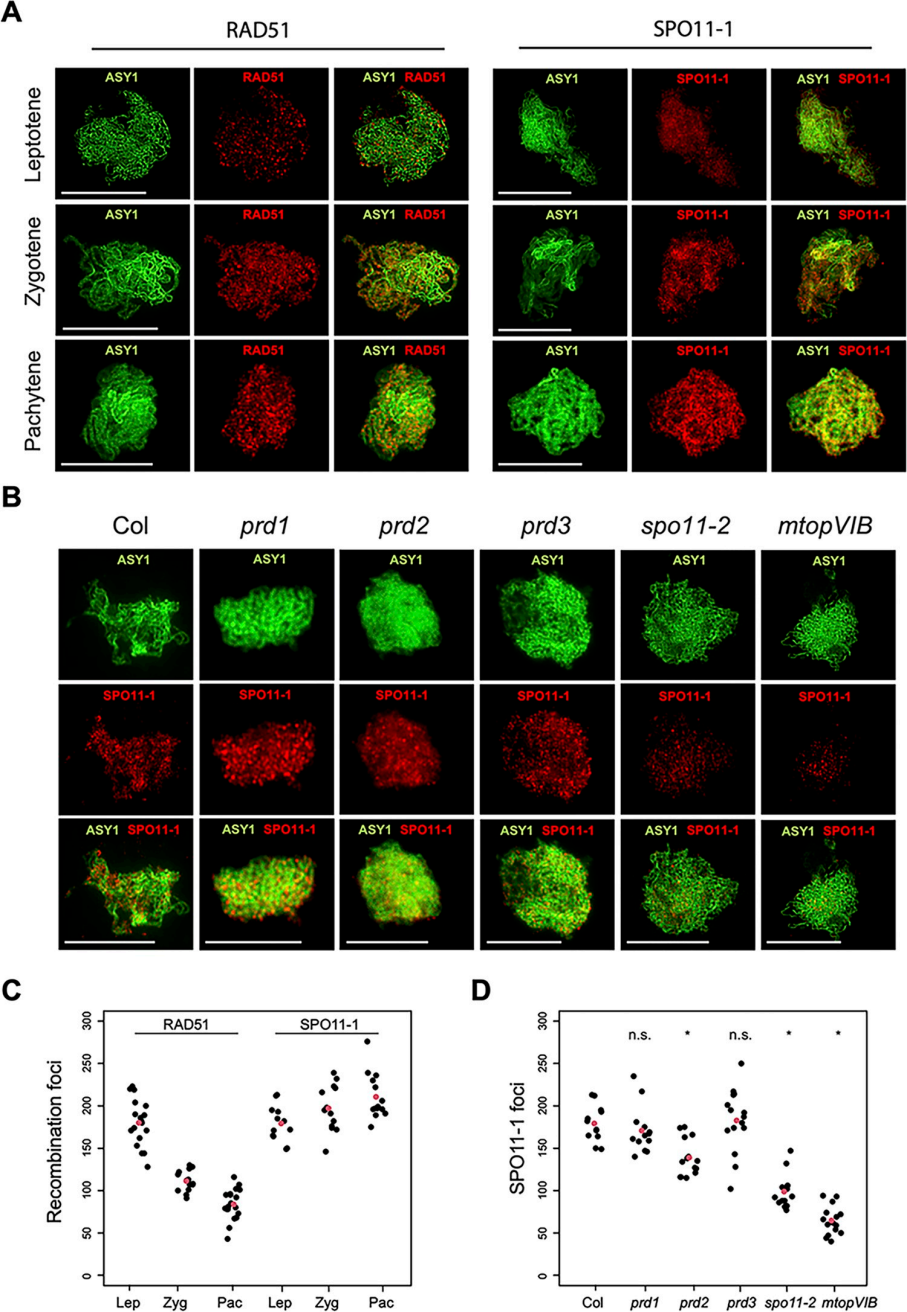
SPO11-1-MYC localisation in wild type and meiotic DSB defective mutants. (**A**) Localisation of RAD51 or SPO11-1-MYC (red) with ASY1 (green) on wild type (Col) male meiocytes, from leptotene to pachytene stages. Scale bars = 10μM. (**B**) Localisation of SPO11-1-MYC (red) and ASY1 (green) in wild type (Col), *prd1*, *prd2*, *prd3*, *spo11-2* and *mtopVIb*, during early prophase I. Scale bars = 10μM. (**C**) Plot showing RAD51 and SPO11-1-MYC foci counts in wild type (Col) at the leptotene (Lep), zygotene (Zyg) and pachytene (Pac) stages of meiosis. Black dots represent individual measurements, and red dots represent mean values. (**D**) Plot showing SPO11-1-MYC foci counts in wild type (Col) and DSB mutants in early prophase I. Black dots represent individual measurements, and red dots represent mean values. A Mann-Whitney-Wilcoxon test was used to compare SPO11-1-MYC foci count between wild type (Col) and mutants. “*” indicates a statistically significant difference. “n.s.” indicates a non-statistically significant difference.

To study the progression of DSB formation during meiosis, we co-immunostained for ASY1 and RAD51 or γH2AX in wild type (Col-0) male meiocyte chromosome spreads at the leptotene, zygotene and pachytene stages (Figs 1 and S1). RAD51 is a recombinase that marks recombination sites, γH2AX is the phosphorylated form of H2AX at residue S139 and accumulates at DSB sites, while ASY1 is a marker of the chromosome axis that becomes depleted on synapsed chromosomes [51]. The distinctive changes in ASY1 immunostaining during meiosis allow the three meiotic stages to be identified (Figs 1A and S1) [51]. As previously reported, we observed that the number of RAD51 and γH2AX foci decreased throughout prophase I, reducing by 53.4% and 59.8%, respectively, at pachytene relative to leptotene stage (Figs 1A and 1C and S1 and S1 Table) [52]. This prompted us to investigate the localisation of SPO11-1 at the same stages. We used a previously characterized epitope-tagged SPO11-1-MYC line [10], and co-immunostained using ASY1 and MYC antibodies. We observed that the mean number of SPO11-1-MYC foci was 179 at leptotene (Fig 1A and 1C). In contrast to RAD51 or γH2AX foci, which progressively decrease from leptotene through pachytene, SPO11-1-MYC foci number increased to 197 at zygotene and 211 at pachytene, representing an increase of 10% and 18%, respectively (Fig 1A and 1C and S1 Table). This suggests that SPO11-1-MYC continues to be recruited to meiotic chromosomes during synapsis at pachytene, unlike the formation of DSB foci which are suppressed.

### SPO11-1 localisation is partially dependent on PRD2, SPO11-2 and MTOPVIB, but not PRD1 or PRD3

To test if the recruitment of SPO11-1 to meiotic chromatin requires other components of the DSB machinery, we immunostained for ASY1 and SPO11-1-MYC in mutants defective in DSB formation, namely *prd1*, *prd2*, *prd3*, *mtopVIB* and *spo11-2* [2,6–8]. SPO11-1-MYC foci associated with chromatin were detected in all mutants (Fig 1B), suggesting that each component of the DSB machinery is not strictly essential to recruit SPO11-1-MYC to meiotic chromatin. However, we observed significant differences in SPO11-1-MYC foci numbers between the mutants (Fig 1B). The localisation of SPO11-1-MYC was not affected in *prd1* (Mann-Whitney-Wilcoxon test (MWW) *P =* 0.192) and *prd3* (MWW, *P =* 0.464), with foci numbers not significantly different to those in wild type (Fig 1B and 1D and S2 Table). In contrast, the number of SPO11-1-MYC foci was reduced to 77.5% in *prd2* (MWW, *P =* 7.5x10^-4^), while they were reduced to 55.2% and 36.1% in *spo11-2* (MWW *P* = 1.3x10^-5^) and *mtopVIB* (MWW *P* = 1.1x10^-7^), respectively (Fig 1B and 1D and S2 Table). A recent study reported that the reduction of SPO11-1-MYC foci was slightly more severe in *spo11-2* than in *mtopVIb*, which we did not observe in our study [53]. However, as we used a different *SPO11-1-MYC* transgenic line this may have contributed to the difference in the phenotype observed. Nevertheless, both studies are concordant in showing that SPO11-1 foci localisation is severely disrupted in *spo11-2* and *mtopvib*. Therefore, PRD1 and PRD3 are not required for SPO11-1-MYC foci formation, while PRD2, SPO11-2 and MTOPVIB support the localisation of SPO11-1-MYC during meiosis. As SPO11-1 forms a catalytic complex with SPO11-2 and MTOPVIB [2], this likely explains the stronger defect in SPO11-1 localisation observed in these mutants.

### PRD3 and SPO11-1 are differentially associated with the chromosome axis protein ASY1 during prophase I

The chromosome axis is essential for DSB formation [33], and we demonstrated that SPO11-1 can localise on the chromatin in a non-active form. We reasoned that the low number of recombination foci on synapsed chromosomes may be caused by the absence of localisation of an auxiliary protein of the DSB complex that preferentially binds to the unsynapsed chromosome axes. As budding yeast Mer2 and its orthologs Asy2 in Sordaria and IHO1 in mouse associate with the chromosome axis [39,54–55], this prompted us to investigate the localisation of PRD3 (an ortholog of Mer2) during prophase I. We epitope-tagged a 5.6 kb *PRD3* genomic clone with 3**×**HA at the C-terminus of the protein, under the control of the endogenous *PRD3* promoter. This construct was used to transform *prd3-3/+* heterozygotes and complementation of homozygous mutants was assessed in the T_2_ generation (S2A–S2C Fig). In homozygous *prd3-3* mutants, chromosomes fail to synapse at pachytene, 10 univalents are detected at metaphase I and chromosome mis-segregation is apparent from anaphase I onwards, which causes sterility (S2A–S2C Fig) [8]. *PRD3-HA prd3-3* tagged lines show restoration of fertility, and no meiotic defects were detected by cytological analysis (S2A–S2C Fig). Thus, PRD3-HA is functional and complements the *prd3-3* phenotype.

**Fig 2:**
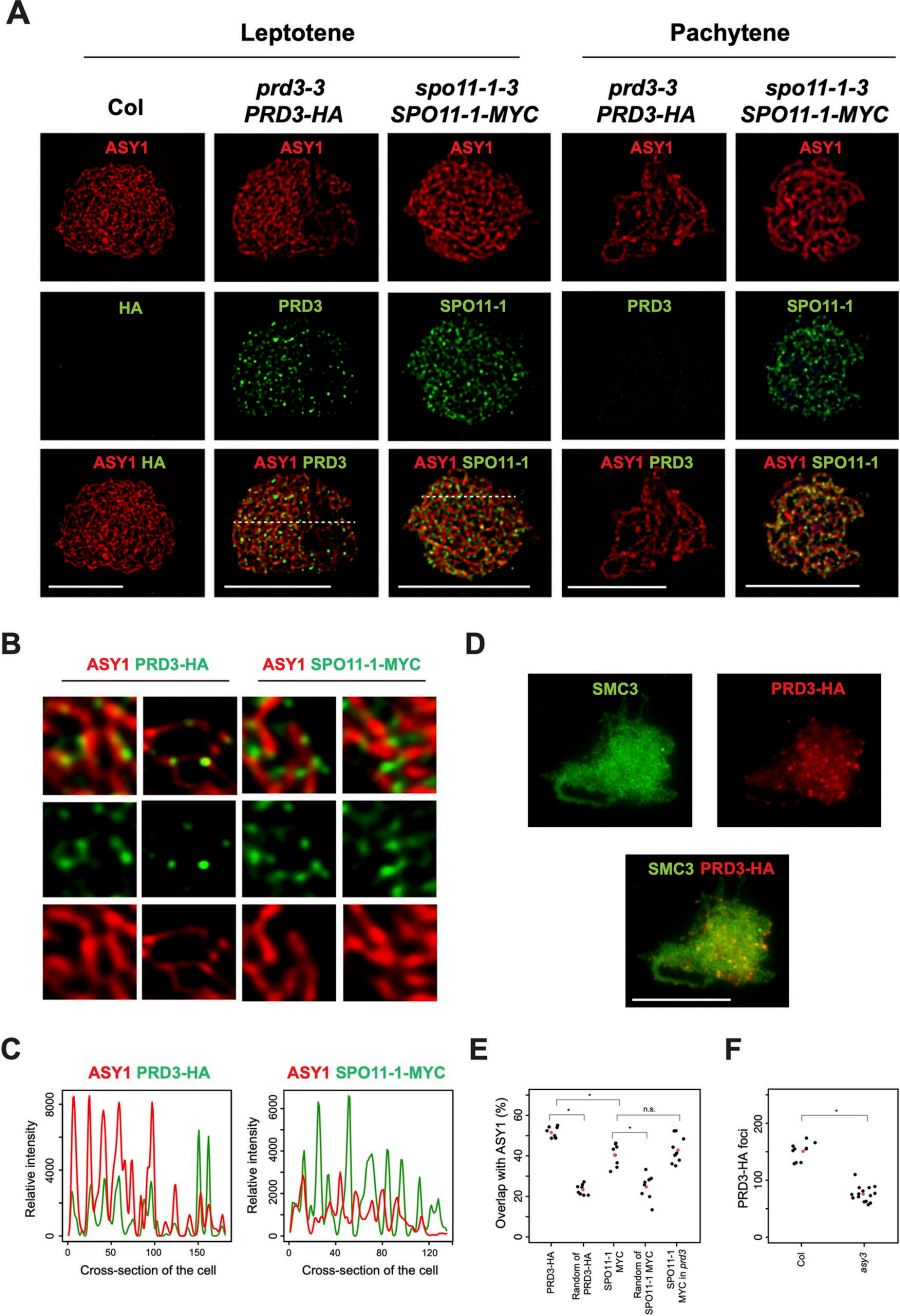
Spatial and temporal localisation of PRD3 in early prophase I of meiosis. (**A**) Localisation of ASY1 (red) and PRD3-HA (green) or SPO11-1-MYC (green) on wild type (Col), *PRD3-HA prd3-3* and *SPO11-1-MYC spo11-1-3* male meiocytes at leptotene and pachytene stages. White dashed lines represent the cross-sections used for the representative co-localisation analysis in Fig 2C. (**B**) Close-up representative images showing the differential co-localisation of PRD3-HA (green) or SPO11-1-MYC (green), in relation to ASY1 (red). (**C**) Representative co-localisation analysis of ASY1 and PRD3-HA or SPO11-1-MYC from the cross-sections of the cells represented with dashed lines in Fig 2A. (**D**) Localisation of SMC3 (green) and PRD3-HA (red) in *asy3* at leptotene stage. (**E**) Plot showing the degree of overlap analysis of PRD3-HA foci with ASY1 or SPO11-1-MYC foci with ASY1 in wild type (Col) or *prd3*. To test for significance, images of PRD3-HA or SPO11-1-MYC were rotated 180 degrees and the degree of co-localisation with ASY1 was calculated (therein called “random of PRD3-HA” and “random of SPO11-1-MYC”). Black dots represent individual measurements, and red dots represent mean values. Mann-Whitney-Wilcoxon tests were performed to test for statistical difference. “*” indicates a statistically significant difference. (F) Plot showing the counts of PRD3-HA foci in wild type (Col) and *asy3* in early prophase I. Black dots represent individual measurements, and red dots represent mean values. A Mann-Whitney-Wilcoxon test was used to test for statistical difference. “*” indicates a statistically significant difference. Scale bars = 10μM.

PRD3-HA expression was detected by immunoblotting in floral bud protein extracts and by immunolocalisation in chromosome spreads from *PRD3-HA prd3-3* male meiocytes (Figs 2A and S2D). We observed that PRD3-HA was localized on the chromatin to form an average of 150.9 foci at leptotene stage (Fig 2A and S3 Table). The detection pattern of PRD3-HA at leptotene is similar to the localisation of SPO11-1-MYC and MTOPVIB (Fig 2A) [2, 10]. However, we observed differences in the localisation of PRD3-HA and SPO11-1-MYC relative to the chromosome axis stained with ASY1 (Fig 2B). We observed that PRD3-HA foci were more frequently overlapping with ASY1, whereas SPO11-1-MYC foci were more frequently adjacent to ASY1 signal (Fig 2B). To quantify this observation, the signal of ASY1 and SPO11-1-MYC or PRD3-HA were compared using SoftWoRx imaging software. Each nucleus was divided into cross-sections of equal size and the information from each section was used to determine the degree of overlap between the two signals (Fig 2C). Using this approach, we observed that PRD3-HA foci co-localized with ASY1 at higher frequency than SPO11-1-MYC (52.7% vs 39.3%, MWW *P =* 1.6x10^-4^) (Fig 2E and S4 Table). To determine the proportion of foci that overlap with ASY1 by random chance, we rotated the images of PRD3-HA and SPO11-1-MYC by 180^o^ and repeated the overlap analysis in relation to ASY1. This analysis indicates that both PRD3-HA and SPO11-1-MYC co-localized with the axis more often than expected through random chance (MWW *P* = 9.3x10^-4^, *P* = 3.1x10^-4^, respectively) (Fig 2E and S4 and S5 Tables). This suggests that PRD3 is more closely associated with the axis than SPO11-1. We also quantified the overlap between SPO11-1-MYC and ASY1 in *prd3* and found no significant difference with wild type (MWW, *P =* 0.573) indicating that PRD3 is not required for the association of SPO11-1-MYC with the axis (Fig 2E and S4 Table).

A further difference in protein localisation was observed at late pachytene, where MTOPVIB and SPO11-1-MYC remain associated with meiotic chromatin, whereas PRD3-HA is no longer detected (Fig 2A) [2,10]. At this stage, the homologous chromosomes are fully synapsed following PCH2-mediated remodelling of the chromosome axis and installation of the SC [51]. This meiotic stage also has fewer RAD51 foci (Fig 1A), as previously reported [52]. Together these observations indicate that although SPO11-1 is abundantly localised on meiotic chromatin at late pachytene, the protein is not functionally active at generating DSBs, potentially due to the absence of PRD3 at this stage.

Our observation that PRD3 closely associates with the chromosome axis reveals a feature of PRD3 that is evolutionary conserved with its orthologs budding yeast Mer2, Sordaria Asy2 and mouse IHO1, which are also associated with the axis [39,54–55]. To further investigate the functional interaction of PRD3 with the axis, we tested if PRD3 localisation is dependent on ASY3. In *asy3*, ASY1 localisation is severely disrupted and DSB formation is defective [28]. Co-staining of PRD3 with SMC3, a component of the cohesin complex marking the chromosome axis, revealed that the number of PRD3 foci was reduced by 49.8% in *asy3* (MWW *P* = 3.6x10^-5^) (Fig 2D and 2F and S3 Table), highlighting a requirement for ASY3 in the localisation of PRD3 for DSB formation.

### PRD3 physically interacts with chromosome axis proteins

**Fig 3:**
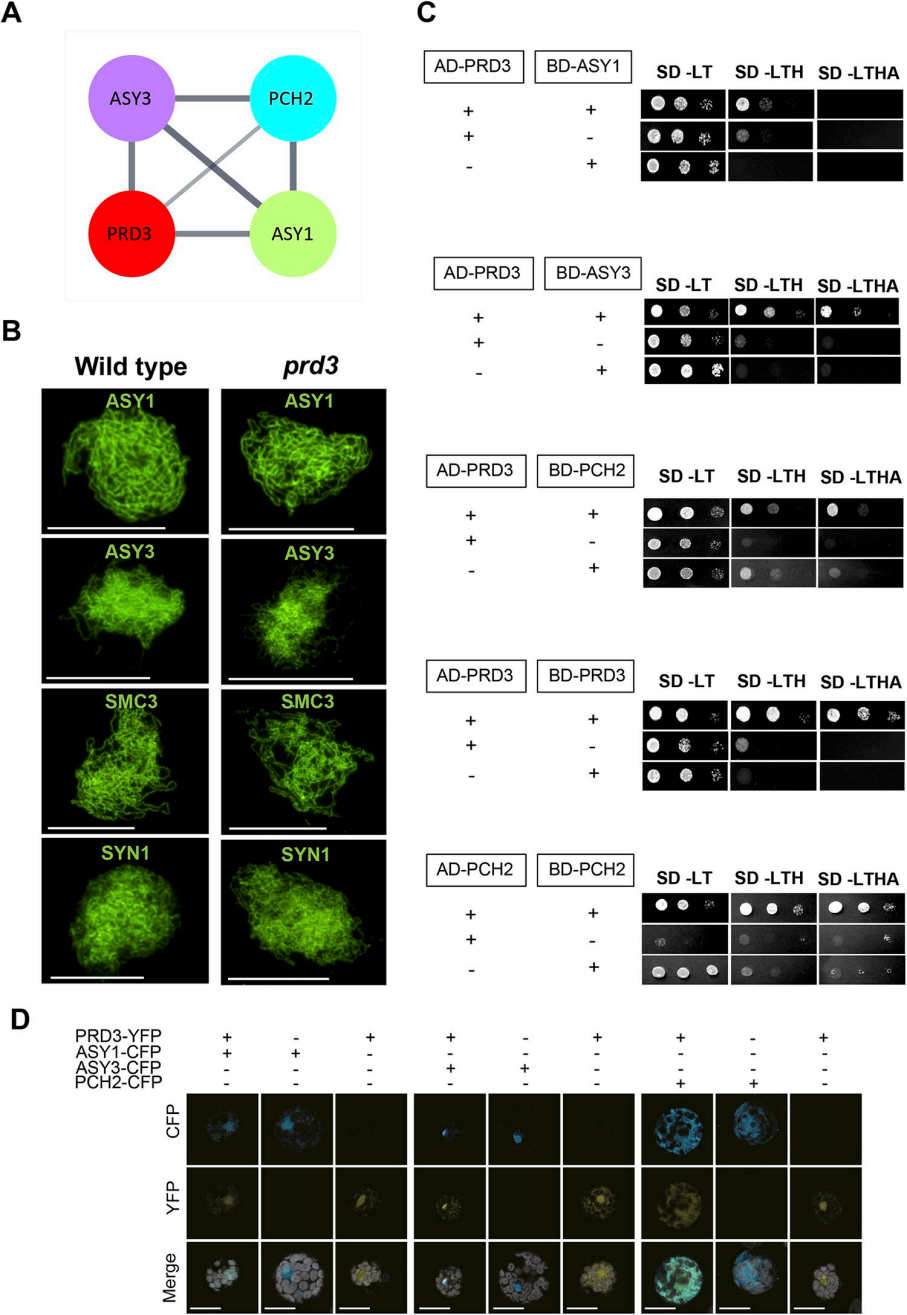
PRD3 interacts with components of the chromosome axis. (**A**) String protein association network showing the linkage between ASY1, ASY3, PCH2 and PRD3. The width of the string represents the strength of the association between the two proteins. (**B**) Immunostaining of ASY1, ASY3, SMC3 and REC8 in wild type and *prd3*. Scale bars = 10 μM. (**C**) Serial drop dilutions (undiluted, 1/10 dilution, 1/100 dilution) of yeast-two hybrid colonies grown on selective media to test the interaction between PRD3, ASY1, ASY3 and PCH2. “+” indicates presence of the Arabidopsis coding sequence in the *AD*- or *BD*-fused vector used for transforming yeast whereas “-”indicates absence of the Arabidopsis coding sequence in the *AD*- or *BD*-fused vector. SD-LT means synthetic defined medium lacking leucine and tryptophan and this medium selects for co-transformants yeast cells. SD-LTH means synthetic defined medium lacking leucine, tryptophan and histidine. SD-LTHA means synthetic defined medium lacking leucine, tryptophan, histidine and adenine. SD-LTH and SD-LTHA test for protein interaction under low- and high-stringency conditions, respectively. (**D**) Co-localisation assay of the fusion proteins PRD3, ASY1, ASY3 and PCH2 in Arabidopsis protoplasts. “+” indicates presence of the coding sequence in the *CFP* or *YFP*-fusion vector during protoplast transfection whereas “-”indicates absence of the coding sequence in the *CFP* or *YFP*-fusion vector. Scale bars = 20μM.

We hypothesised that the reduced number of RAD51 foci between early and late prophase may be linked to the remodeling of the chromosome axis, and the progressive synapsis taking place between homologous chromosomes in late prophase I [51]. To identify components of the DSB machinery that may interact with the chromosome axis, we revisited data from an ASY1-affinity immunoprecipitation pull-down assay from isolated Brassica meiocytes, which is closely related to Arabidopsis. We found that PRD3 was the only known component of the DSB machinery to be immunoprecipitated with ASY1 (S6 Table) [32]. Moreover, peptides for PRD3 were found in the two-independent ASY1 pull-down experiments that have the highest recovery for ASY3 peptides, which is another component of the chromosome axis. [32]. The proteins identified from co-IP-MS were searched against the Arabidopsis TAIR10 proteome to identify orthologues using BLAST. This resulted in 482 proteins and the proteins were used as seeds to query the STRING database version 11.5 [56], which returned a total of 7,124 interactions. Among this network, PRD3 forms an interacting sub-network with ASY1, ASY3 and PCH2 (Fig 3A).

To confirm a physical interaction between Arabidopsis PRD3, ASY1 and ASY3, we used the GAL4 yeast two-hybrid system in budding yeast. Consistent with the Brassica affinity proteomic data, we observed that PRD3 interacts with ASY1 on the less stringent medium SD -LTH, but not on the most stringent medium SD -LTHA, which may suggest weak or transient interaction between the two proteins in this heterologous system (Fig 3C). In contrast, PRD3 strongly interacts with ASY3 on both SD -LTH and SD -LTHA media (Fig 3C). We also tested the interaction between PRD3 and PCH2, since PCH2 is recruited to the axis when chromosomes are synapsing and remodels the axis through protein-protein interactions [57]. We observed that yeast co-transformed with PRD3 and PCH2 were able to grow on both SD -LTH and SD -LTHA selective media. Yeast transformed with BD-PCH2 also grew on these media, but at a lower rate, which suggests that PRD3 can interact with PCH2 in a yeast two-hybrid assay (Fig 3C). Lastly, we observed that PRD3 and PCH2 can self-dimerise as yeast co-transformed with AD-PRD3 and BD-PRD3 or AD-PCH2 and BD-PCH2 grew on the restrictive medium (Fig 3C). Additionally, we noted that yeast transformed with AD-PCH2 were slow to grow on SD-LT (Fig 3C), suggesting that the expression of this heterologous protein causes some toxicity to the yeast cells.

To further test the interaction between PRD3 and ASY1, ASY3 or PCH2 in Arabidopsis, the proteins were fused to YFP or CFP fluorescent proteins and transiently expressed in protoplasts. Expression of PRD3-YFP showed that the protein mainly localises in the nucleus (Fig 3D). Co-expression of PRD3-YFP and ASY1-CFP or PRD3-YFP and ASY3-CFP revealed extensive overlap of the two fluorescent signals in the nuclei, indicating a propensity of the two proteins to co-localise in the nucleus. In contrast, it appeared that PCH2-CFP localised in both the cytoplasm and nucleus, which is in accord with its localisation in meiotic cells [58]. The expression of PRD3-YFP in the presence of PCH2-CFP shows that PRD3-YFP is found localising in these two cellular compartments and the fluorescent signal overlaps with the signal of PCH2-CFP (Fig 3D). Overall, our data suggest that PRD3 can interact with components of the chromosome axis consistent with the co-localisation of PRD3 with ASY1 on the Arabidopsis meiotic chromosome axes.

### The chromosome axis is required for DSB formation, but not for SPO11-1 localisation

**Fig 4:**
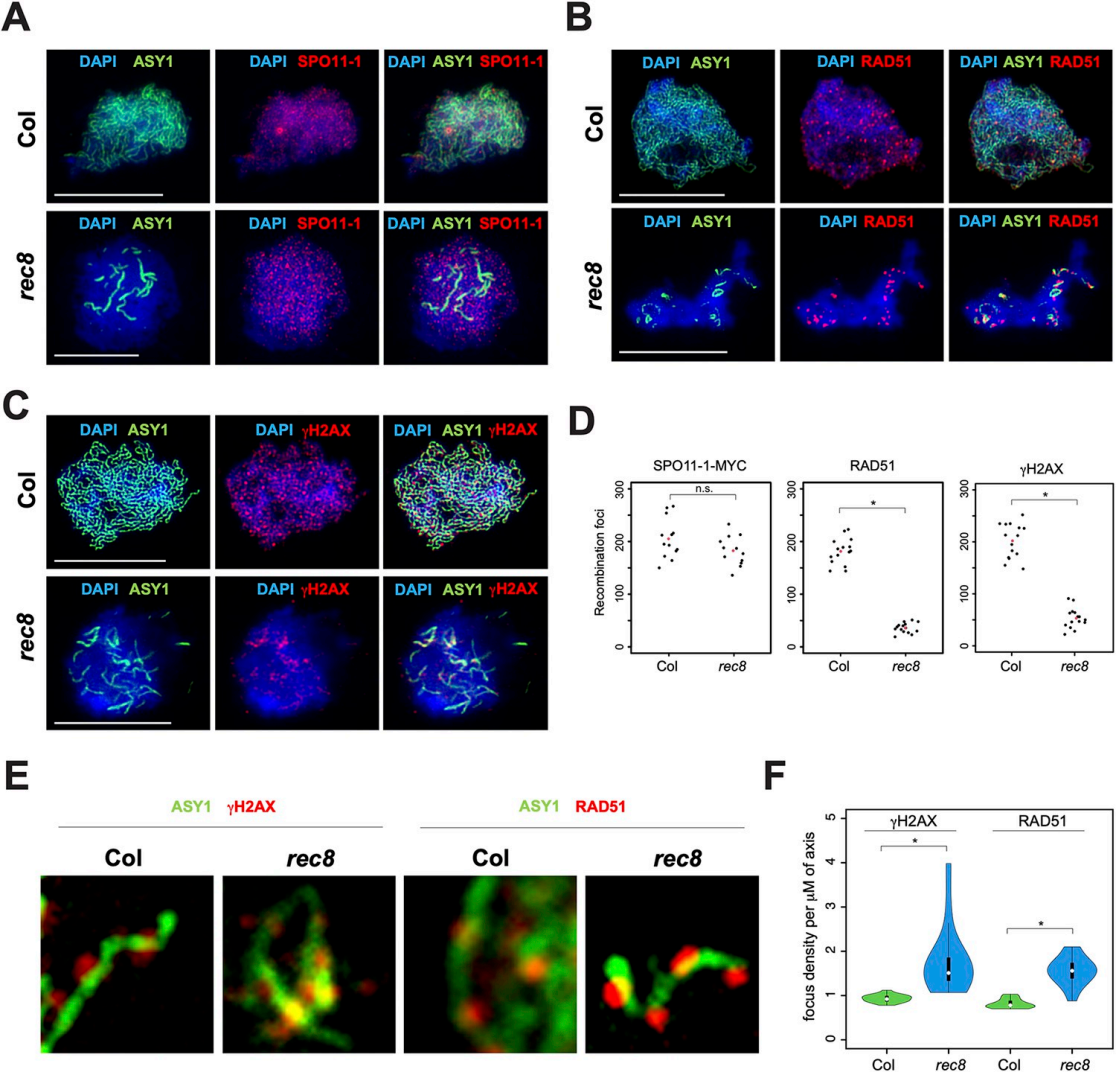
SPO11-1, RAD51 and γH2AX staining in wild type and *rec8*. (**A**) Staining of SPO11-1-MYC (red) and ASY1 (green) in wild type (Col) and *rec8*. Chromatin is stained with DAPI (blue). Scale bars = 10 μM. (**B**) Staining of RAD51 (red) and ASY1 (green) in wild type (Col) and *rec8*. Chromatin is stained with DAPI (blue). Scale bars = 10 μM. (**C**) Staining of γH2AX (red) and ASY1 (green) in wild type (Col) and *rec8*. Chromatin is stained with DAPI (blue). Scale bars = 10 μM. (**D**) Plot showing counts of SPO11-1-MYC, RAD51 or γH2AX foci in wild type (Col) and *rec8*. Black dots represent individual measurements, and red dots represent mean values. Counts of RAD51 and γH2AX foci are taken from Lambing et al. 2020. A Mann-Whitney-Wilcoxon test was used to compare SPO11-1-MYC or RAD51 foci count between wild type (Col) and *rec8*. “*” indicates a statistically significant difference. “n.s.” indicates a non-statistically significant difference. (**E**) Co-staining of ASY1 (green) with RAD51 or γH2AX (red) in early prophase I in wild type (Col) and *rec8*. (**F**) Violin plot representing the distribution of RAD51 or γH2AX focus density per μm of axis between nuclei in wild type (Col) and *rec8*. A Mann-Whitney-Wilcoxon test was performed to test for significance. “*” indicates a statistically significant difference.

To investigate the impact of altering the structural integrity of the chromosome axis on DSB formation, we repeated ASY1 immunostaining with RAD51, or ASY1 with γH2AX, or ASY1 with SPO11-1-MYC in *rec8*. In *rec8*, only short stretches of axis are formed and the numbers of RAD51 and γH2AX foci are significantly reduced (Fig 4B and 4D), as previously reported [33]. Further examination revealed that most RAD51 and γH2AX foci were closely associated with the short axis stretches formed in *rec8*, while only 8% of RAD51 foci (n = 10) and 13% of γH2AX foci (n = 10) were overlapping with the DAPI-stained chromatin that lacks an axis (Fig 4B). In contrast, the total number of SPO11-1-MYC foci was only slightly reduced in *rec8* (Fig 4A and 4D and S7 Table) (205.2 vs 182.6; MWW *P* = 0.149), and the foci were observed throughout chromatin and not always on the short stretches of axis (Fig 4A). Overall, this indicates that the localisation of SPO11-1 on meiotic chromatin does not require the presence of an axis, but rather the axis is required for the formation of DSBs along chromosomes. Additionally, we observed that RAD51 and γH2AX foci tend to cluster on the short stretches of axis that form in *rec8* (Fig 4D). To quantify this observation, we counted the number of RAD51 and γH2AX foci on the axis, and measured the length of the axis. We found that the density of RAD51 and γH2AX foci per μm of axis were significantly increased by 1.9-fold in *rec8* compared to wild type (MWW *P* = 7.4x10^-6^, *P* = 4.1x10^-6^) (Fig 4E and 4F and S8 Table).

**Fig 5:**
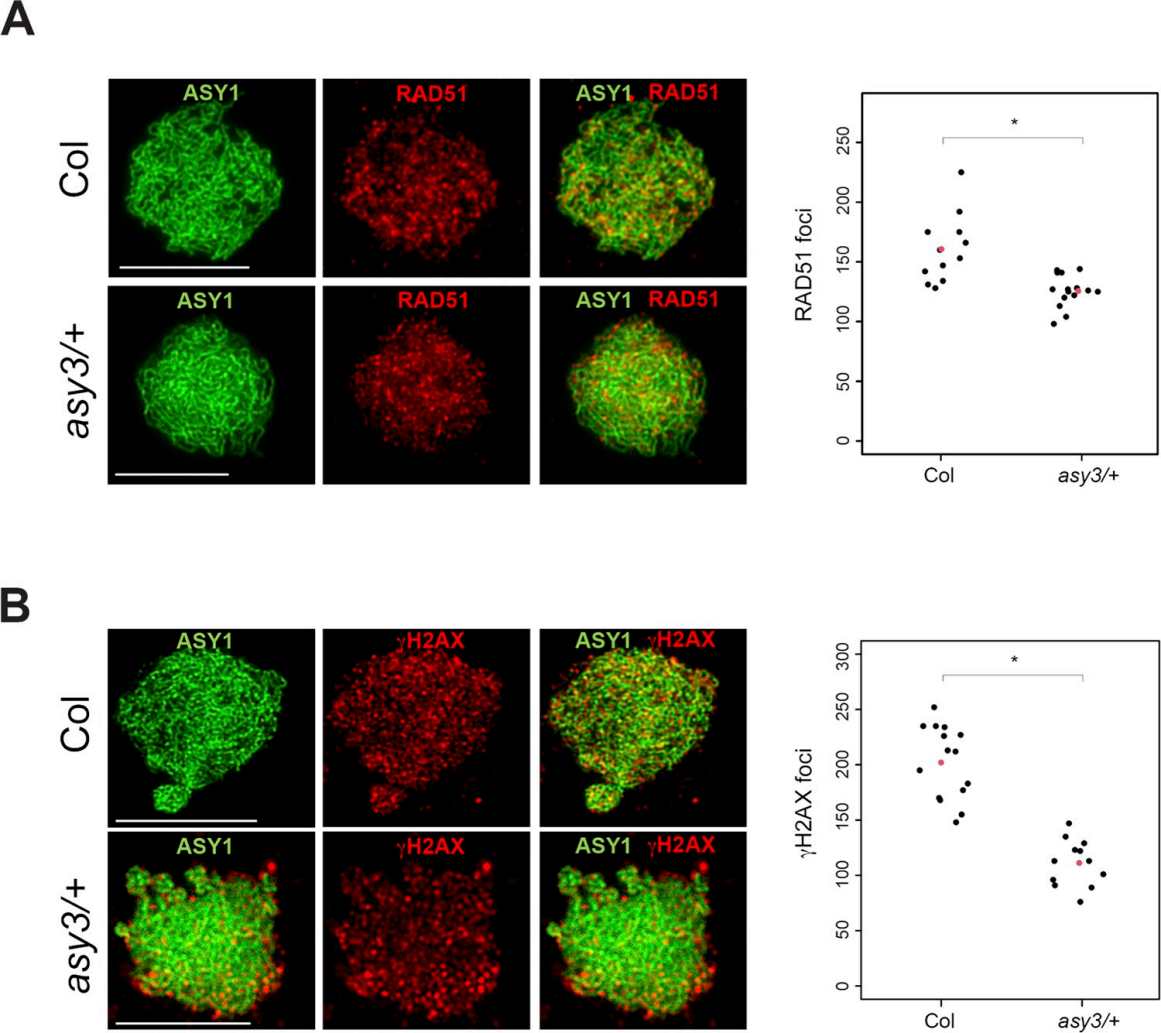
*ASY3* gene copy number influences DSB formation. (**A**) Co-staining of ASY1 (green) and RAD51 (red) in wild type (Col) and *asy3/+* heterozygotes. Plot showing counts of RAD51 foci in wild type (Col) and *asy3/+*. Black dots represent individual measurements, and red dots represent mean values. A Mann-Whitney-Wilcoxon test was used to compare counts of RAD51 foci between wild type (Col) and *asy3/+*. “*” indicates a statistically significant difference. (**B**) Co-staining of ASY1 (green) and γH2AX (red) in wild type (Col) and *asy3/+* heterozygotes. Plot showing counts of γH2AX foci in wild type (Col) and *asy3/+*. Black dots represent individual measurements, and red dots represent mean values. A Mann-Whitney-Wilcoxon test was used to compare counts of γH2AX foci between wild type (Col) and *asy3/+*. “*” indicates a statistically significant difference.

Our data indicate that the linear integrity of the chromosome axis is important for meiotic DSB formation, but it is unclear if a change in the level of axis proteins on the linear structure can also influence DSB formation. A recent study showed that in *asy3/+* heterozygotes, the underlying chromosome axis and homologous pairing are similar to wild type, but the level of axis proteins on the chromosomes is reduced and the crossover landscape is distalized [59]. However, the effect on DSB formation in *asy3/+* heterozygotes was not reported. Therefore, we immunostained ASY1 and RAD51 or γH2AX in *asy3/+* to investigate effects on DSB formation. We observed a significant reduction of RAD51 and γH2AX foci in *asy3-1/+* compared to wild type (MWW test *P =* 2.5x10^-4^, *P =* 1.3x10^-5^, respectively) (Fig 5A and 5B and S9 Table). Altogether, these data show that the structural integrity of the axis and its protein composition influence DSB formation.

### ZYP1 nucleation occurs in *prd3* but is absent in *spo11-1*

Since PRD3 can interact with components of the chromosome axis, we next tested if PRD3 is required for axis formation. We immunostained for the axis components ASY1 and ASY3, and the cohesin subunits SMC3 and REC8 in wild type and *prd3*. In wild type, all four proteins form a linear signal along the chromosomes in early prophase I (Fig 3B). In *prd3*, the localisation of all axis proteins appeared normal, which is consistent with the axis assembling independently of PRD3 and DSB formation (Fig 3B).

**Fig 6:**
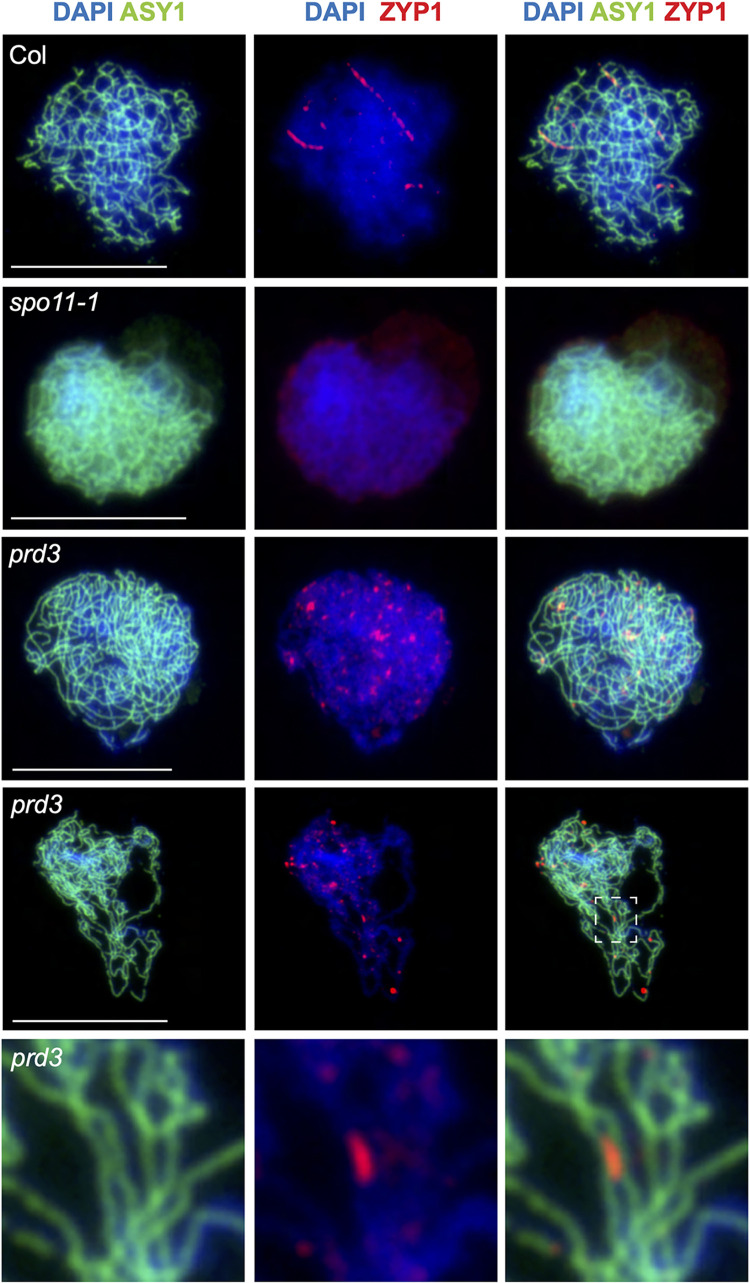
ZYP1 synapsis installation in wild type and *prd3*, but not in *spo11-1*. Staining of ASY1 (green), ZYP1 (red) and chromatin (DAPI, blue) in wild type (Col), *prd3* and *spo11-1*. The dashed white box represents a region where ZYP1 forms a short stretch between two co-aligned ASY1-stained axes. The close-up image is shown underneath. Scale bars = 10 μM.

In budding yeast and *Coprinus cinereus*, SPO11 has additional functions besides DSB formation [60, 61]. In budding yeast, SPO11 has a role during pre-meiotic S-phase, and homolog pairing is partially dependent on this function, while SC nucleation is partially restored in *Coprinus cinereus spo11* mutants, only when premeiotic DNA replication does not occur [60,61]. Hence, to investigate if Arabidopsis SPO11-1 and PRD3 exhibit a separation of function in supporting ZYP1 localisation, we immunostained ASY1 and ZYP1 in wild type, *prd3* and *spo11-1*. In wild type, ZYP1 starts to nucleate during zygotene, where it forms foci and short stretches representing the initiation of synapsis between chromosomes (Fig 6). In *spo11-1*, most nuclei showed a complete absence of ZYP1 localisation, as previously reported [50], and we only rarely observed nuclei with a few ZYP1 foci (14.3%, 2 out of 14 nuclei) (Fig 6). In contrast, we found that most nuclei in *prd3* (89.5%, 17 out of 19) showed some degree of ZYP1 localisation (Fig 6). In some instances, we observed numerous ZYP1 foci and short stretches that co-localise with the ASY1-stained axis (Fig 6). We also observed some stretches of ZYP1 between two co-aligned ASY1-stained axes (Fig 6). Overall, these indicate that PRD3 and SPO11-1 play distinct functions in the recruitment of ZYP1 to meiotic chromatin that is independent of DSB formation. These also show that chromosome co-alignment and synapsis initiation can occur, albeit only partially, in the absence of meiotic DSBs in Arabidopsis.

### Meiotic progression is not delayed in the absence of DSB formation

The progression of meiosis is tightly linked with the formation of DSB and crossovers in budding yeast and mice [45,46,62]. In these species, the absence of DSBs or crossovers leads to the activation of a prophase checkpoint causing the arrest of meiosis or apoptosis [45,46,62]. In Arabidopsis, meiosis is delayed in mutants proficient in DSB formation but defective for synapsis and crossover formation [50,51,63–65]. A recent study showed that the duration of prophase I is also prolonged in Arabidopsis *spo11-1* [66]. In budding yeast, SPO11 has been implicated in the duration of pre-meiotic S-phase and homolog pairing, beside its function in DSB formation [60]. Since SPO11-1 and PRD3 show a separation of function in the nucleation of ZYP1, we asked if meiotic duration is delayed in *prd3*, as was reported in *spo11-1*.

**Fig 7:**
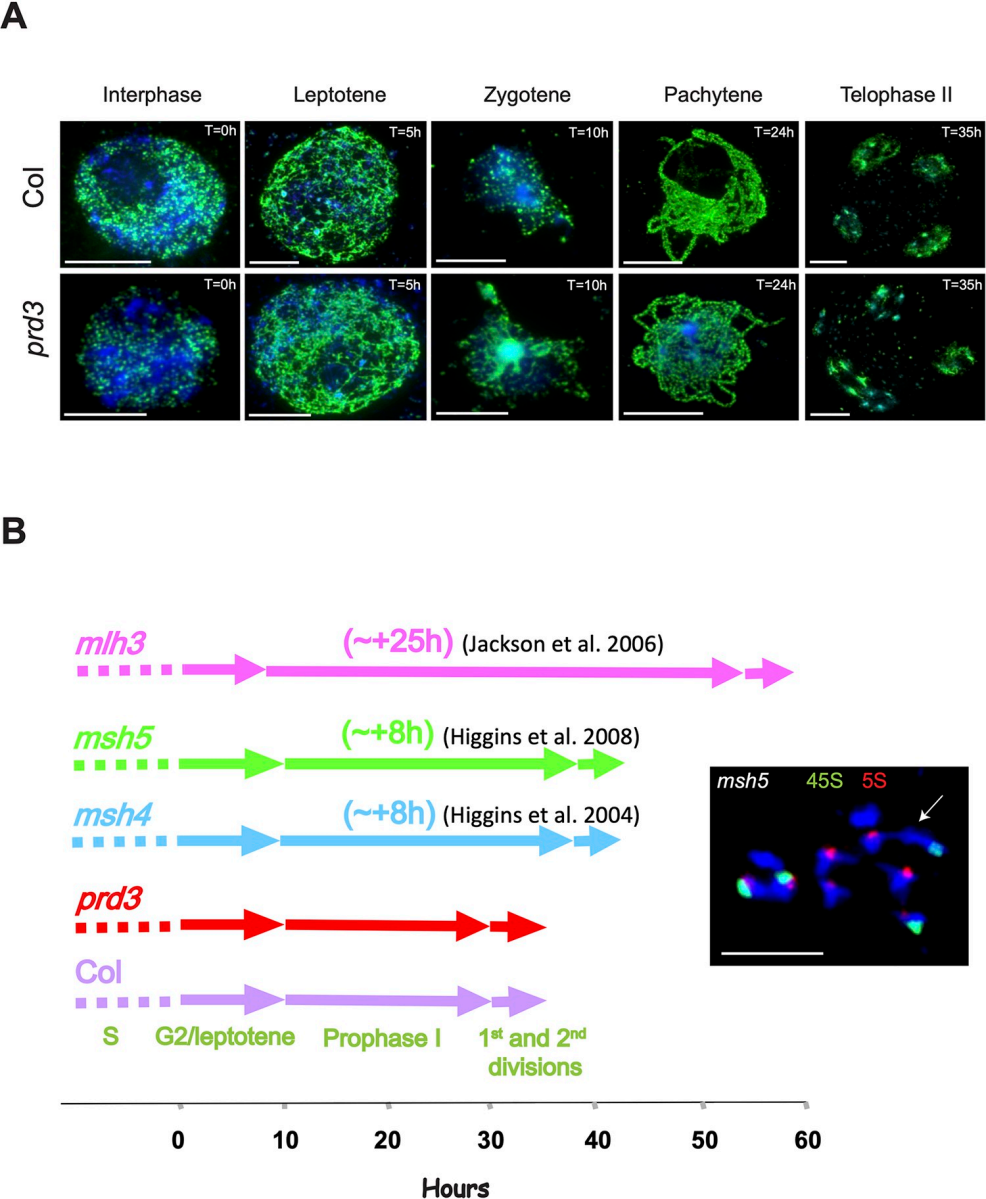
Meiotic duration in wild type and *prd3* measured using BrdU incorporation. (**A**) Staining of BrdU (green) and chromatin (DAPI, blue) at defined pre-meiotic and meiotic stages following a BrdU pulse in wild type (Col) and *prd3*. Scale bar = 10 μM. (**B**) Schematic representation showing the duration of meiosis in wild type (Col), *prd3*, *msh4* [63], *msh5* [64] and *mlh3* [65]. Metaphase I labelled with *45s* (green) and *5s* (red) rDNA FISH probes. White arrow shows a chiasma between non-homologous chromosomes in *msh5*. Chromatin is stained in blue. Scale bar = 10 μM.

To measure the duration of meiosis, we performed a bromodeoxyuridine (BrdU) pulse-chase labelling of pre-meiotic S-phase nuclei followed by a time course experiment to determine the duration of meiotic stages in wild type and *prd3*. In wild type, the first appearance of BrdU-labelled leptotene and zygotene nuclei occurred at 5 and 10 hours post pulse, respectively, while pachytene cells were first labelled at 24 hours and telophase II cells at 35 hours post-BrdU pulse (Fig 7A and 7B and S10 Table). These observations are consistent with previous reports [67], and indicate that the period between pre-meiotic DNA replication and telophase II is approximately 35 hours in wild type. In *prd3*, homologous chromosomes fail to synapse due to the absence of DSBs and therefore cells cannot be classified as zygotene or pachytene based on the establishment of the synaptonemal complex [68]. However, in Arabidopsis, zygotene and pachytene stages can also be identified based on their degree of chromatin compaction and chromocenter clustering [69]. Hence, we used these criteria for the analysis, and we observed no difference in meiotic duration in *prd3* (Fig 7A). BrdU-labelled leptotene nuclei were detected at 5 hours in *prd3*, pachytene-like nuclei at 24 hours and telophase II nuclei at 35 hours post pulse, which was similar to wild type (Fig 7A and 7B and S10 Table). Altogether, these data indicate that Arabidopsis meiotic cells progress through meiosis with normal duration in the absence of PRD3 and DSBs. This also suggests that Arabidopsis lacks a prophase I checkpoint that triggers a delay or the arrest of meiosis in the absence of DSB formation.

Previous meiotic time course experiments based on BrdU-pulse chase were reported in 4 mutants with varying delays in prophase I [51,63–65]. In *msh4* and *msh5* mutants, prophase I was found to be prolonged by 8 hours, while in *mlh3*, it was prolonged by 25 hours (Fig 7B) [51,63–65]. In *pch2*, a time course experiment was conducted up to 36 hours post-BrdU pulse and revealed that the labelled meiotic nuclei were at zygotene stage, which indicates a delay of at least 5–8 hours, relative to wild type [51]. MSH4 and MSH5 are involved in the stabilisation of joint molecule structures and MLH3 stimulates the cleavage of these structures and their resolution into crossovers, whereas PCH2 remodels the chromosome axis to influence the recombination outcome [51,65,70]. Since the progression of prophase I is not delayed in the absence of recombination in *prd3*, we speculated that the prolonged prophase I stage occurs in response to aberrant recombination events. These events are difficult to detect cytologically at pachytene stage but are more easily detected at metaphase I when the chromosomes are aligned on the metaphase plate. This stage occurs later than pachytene stage, but we reasoned that not all aberrant recombination events will be resolved at pachytene and that some events may persist until metaphase I similar to the report of non-homologous chromosome connections detected in *recq4a* metaphase I nuclei [71]. Hence, to investigate if the delay in meiosis progression was due to aberrant recombination, we analysed chromosome spreads of *msh5* metaphase I nuclei and labelled *45S* and *5S* rDNA with fluorescent probes (Fig 7B). We observed that 4% of nuclei (2 out of 50) showed a bivalent between non-homologous chromosomes in *msh5* and we never observed non-homologous interaction in wild type metaphase I nuclei (n = 100) (Fig 7B). These non-homologous chiasmata are unlikely to be resolved at pachytene, which explains their persistence until metaphase I, whereas aberrant recombination events leading to non-crossovers are likely resolved earlier during the extended prophase I. Altogether, our data suggest that the delay in meiotic progression may occur in response to aberrant recombination events, rather than the activation of a checkpoint in response to the absence of DSBs or synapsis between chromosomes.

## Discussion

Our study provides functional insight into the control of DSB formation during meiosis. We demonstrate that SPO11-1 accumulates on the chromatin throughout prophase I and that its localisation does not always correlate with the presence of DSB markers, including RAD51 and γH2AX. Specifically, SPO11-1 foci number is highest at pachytene, while the number of foci for DSB markers is at a minimum by this stage. Previous work has shown that MTOPVIB is also enriched on the chromatin in late prophase [2], but it remains to be determined if MTOPVIB and SPO11-1 are co-localised at this stage. However, since MTOPVIB is required for the recruitment of SPO11-1 [53], it is likely that the reduction of DSB markers at pachytene is not due to a change in the interaction between SPO11-1 and MTOPVIB. In contrast, our cytological investigations revealed that PRD3 localisation mirrors the trend of DSB markers with the highest number of PRD3 foci at leptotene stage, followed by a decrease in foci number throughout the rest of prophase I. At pachytene stage when the chromosomes are fully synapsed, PRD3 is no longer observed associated with meiotic chromatin. At this stage, RAD51 and γH2AX foci numbers are low, which likely represents the final pool of DSBs being processed by the homologous recombination pathway. During the preparation of the manuscript, a separate study also showed that PRD3 is absent from the synapsed chromosomes [53], corroborating our observations. We propose that the temporal differences in localisation between the different components of the DSB machinery dictates the timing of DSB formation, and that the absence of PRD3 from the synapsed chromosomes is sufficient to prevent DSB formation at later stages, despite the presence of SPO11-1 and MTOPVIB. The localisation of SPO11-1 in Arabidopsis differs from that in maize where the number of SPO11-1 foci is at a minimum at pachytene [72]. Maize and Arabidopsis have very different genome size (~2,400 Mbp and 130 Mbp, respectively) and architecture resulting in different physical sizes of their chromosomes and overall axis length (2,824 μM and 220 μm, respectively) [51,72–74]. These varying parameters could influence SPO11-1 localisation in relation to the axis between species.

PRD3 was found as an interacting protein in an immunoprecipitation pull-down assay of the ASY1 axis protein in Brassica [32], and we provide further support that PRD3 is associated with the axis. First, PRD3 physically interacts with ASY1, ASY3 and PCH2 in yeast two-hybrid assays and co-localises with all three axis proteins when transiently expressed in Arabidopsis protoplasts. Second, PRD3 foci co-localise with ASY1 on meiotic chromosomes at a higher frequency than SPO11-1, and higher than random. Third, the localisation of PRD3 is reduced in axis mutants, whereas SPO11-1 and MTOPVIB localisation are normal [53]. Moreover, evolutionary studies in diploid and tetraploid *Arabidopsis arenosa* have found that *PRD3*, along with several axis protein-coding genes, were under selection following an increase in ploidy [75]. This may reflect the adaptive stabilisation of meiosis after a change in ploidy, which suggests co-evolution of a functionally interacting network of proteins. PRD3 shows amino-acid homology with budding yeast Mer2, mouse IHO1 and Sordaria Asy2 [39,54,55]. All three proteins are associated with the axis, revealing a conservation of function during meiosis across kingdoms [39,54,55]. In budding yeast, the chromatin is organised in arrays of loops associated with the axis and SPO11 is bound to the loops, while Mer2 is bound to the axis [39]. Several studies suggest that interaction of the chromatin loops with the axis is a pre-requisite for DSB formation [39,43,44]. We observed that SPO11-1 foci are mostly co-localising with chromatin and not the axis. This is consistent with the negative correlation observed at the fine-scale between SPO11-1-oligos and REC8 ChIP-seq data [33], and provides evidence that aspects of the organisation of the DSB machinery on meiotic chromatin are conserved between Arabidopsis and budding yeast.

The control of meiotic DSB formation is not fully understood in Arabidopsis. DSB formation is reduced in the axis mutants *asy3* and *rec8*, whereas it is increased in the kinase mutant *atm* [28,33,49]. SPO11-1 is localised throughout the chromatin in *rec8*, but DSB markers are mostly in contact with the short stretches of axis found in this mutant. In addition, *ASY3* gene copy number influences DSB formation, and PRD3 localisation is reduced in *asy3* mutants [53]. We propose that the axis forms a scaffold onto which loop-axis interactions promote the formation of SPO11-1-dependent DSBs. Although PCH2 can interact with PRD3, and PCH2 is recruited onto the synapsed chromosomes, it remains unclear if PCH2 is actively involved in the removal of PRD3 at pachytene. The number of recombination markers appears to decrease in *pch2* as meiosis progresses, as observed in wild type and there is no evidence that the number of DSBs is affected in *pch2* [51]. In budding yeast, a first wave of DSBs is formed and activates a pathway dependent on Tel1, the ortholog of ATM, which represses the formation of further DSBs [76]. Tel1 and ATM phosphorylate proteins at [S/T]Q sites [77,78]. Among the known components of the DSB complex in Arabidopsis, only PRD1 and PRD3 are enriched in [S/T]Q sites (S11 Table). PRD3 is particularly interesting because it has 10 SQ/TQ motifs, among which 9 are clustered in a region of 72 amino acids in the N-terminus. In budding yeast, Tel1 phosphorylates Rec114 to repress its interaction with chromatin and inhibits DSB formation [76]. A complete understanding of PRD3 removal from the synapsed regions will require further investigation of how the axis is remodelled during synapsis, and to identify targets of ATM kinase during meiosis.

In budding yeast and mouse, meiotic progression is linked with recombination [45,46,62]. We show that Arabidopsis lacks a checkpoint that responds to the absence of recombination, since meiosis is not delayed when DSB formation is abolished in *prd3*. Previous studies reported that meiotic progression is delayed in Arabidopsis mutants defective for synapsis and crossover formation [63–65]. However, we demonstrated that synapsis and crossovers are not a requirement for meiotic progression. Instead, we propose that abnormal recombination events, including non-homologous connections, are more likely responsible for the delay in meiotic progression. We also uncovered a new function of SPO11 in the deposition of ZYP1, beside its role in DSB formation. We demonstrate that chromosome co-alignment and synapsis can partially occur in the absence of meiotic DSBs, and this is dependent on SPO11-1, but not PRD3. Interestingly, the duration of prophase I was reported to be slightly extended in Arabidopsis *spo11-1* [66]. It is possible that meiotic progression is linked with initial chromosome co-alignment that is dependent on SPO11-1 but independent of DSB formation. Further study will be required to determine if these functions of SPO11-1 require its catalytic activity.

## Materials and methods

### Plant materials

*Arabidopsis thaliana* plants were grown under long day conditions (16 hours light/8 hours dark, 150 μmol light) with 60% humidity at 20°C. The following mutant alleles in the Col-0 accession were used: *spo11-1-4* (WiscDsLox_461-464J19) [32], *spo11-2-3* (GABI_749C12) [6], *prd1-2* [7] (Salk_024703), *prd2-6* (Sail_94_D08) [8], *prd3-3* (GABI_677D06) [8], *mtopVIB-2* (GABI_314G09) [2], *rec8-1* (Salk_091193) [28], *asy3-1* (Salk_143676) [28] and *msh5-1* (Salk_110240) [64]. Lines were obtained from NASC. *SPO11-1-Myc spo11-1* transgenic line was previously reported [10].

### Generation of PRD3-3xHA transgenic lines

A region of 5,637bp corresponding to *PRD3* locus up to the stop codon and including 1.5 kb region upstream of *PRD3* was PCR amplified from Col genomic DNA using Phusion High Fidelity DNA Polymerase. The 3’ end of the amplicon was ligated to the coding sequence of 3xHA to produce PRD3 protein fused to a C-terminus HA epitope tag. The ligation product was cloned into pGreen0229 and transformed into *Agrobacterium tumefaciens* GV3101. Arabidopsis *prd3-3/+* plants were transformed by floral dipping using *Agrobacterium*. Primary transformants were selected on BASTA-supplemented MS medium and the presence of the transgene was confirmed by PCR genotyping using the following primers: AGGAAACTGAAAGGATTCTGAG and ATGCATTACATGTTAATTATTACATGC.

### Western blotting of epitope-tagged PRD3-HA

One gram of unopened flower buds from untagged Col and *PRD3-HA prd3-3* plants were ground in liquid nitrogen then transferred to a solution of Lysis Buffer (25 mM Hepes-NaOH pH 7.9, 5 mM EDTA, 2% SDS, 1 mM PMSF, 2 mM DTT, 1xprotein cocktail inhibitor (Roche, 11873580001)) and incubated for 20 minutes at 95°C. The solution was centrifuged at 13,300 rpm for 20 minutes at 4°C. The supernatant was recovered and diluted with 3 volumes of Dilution Buffer (14 mM Tris pH 8.0, 1% Triton X-100, 150 mM NaCl). Protein extracts and eluted protein solutions were loaded with 1×Laemmli protein loading buffer on a NuPage 3–8% Tris acetate gel (Invitrogen, EA0375BOX). After proteins were resolved using gel electrophoresis, proteins were transferred to a PVDF membrane (Bio-Rad, 162–0177) in a transfer solution (25 mM Tris base, 192 mM glycine, 20% v/v methanol, pH = 8.3) for 1 hour at 100 mV at 4°C. After transfer, the membrane was rinsed with TBST (20 mM Tris base, 150 mM NaCl, pH = 7.6) and incubated in blocking buffer (5% w/v non-fat dried milk in TBST) overnight at 4°C. The membrane was then washed 1×15min and 2×5min with TBST at room temperature. A solution of primary antibody diluted in blocking buffer was added (HA: Roche, 3F10, dilution 1:500) and the membrane was incubated for 2 hours at room temperature. After this, the membrane was washed 1×15min and 2×5min with TBST. A solution of secondary antibody diluted in blocking buffer (HA: anti-rat IgG-HRP, Santa Cruz Biotechnology, sc-2006, dilution 1:4800) was added, and the membrane was incubated for 1 hour at room temperature. The membrane was washed 1×15 minutes and 2×5 minutes with TBST at room temperature. The signal was detected on film with ECL prime Western Blotting detection reagents (GE Healthcare, RPN2232) using a Xograph compact X4.

### Fluorescent-tagged protein co-localization assay in Arabidopsis protoplasts

Protoplast transient expression vectors were constructed using Golden Gate cloning, as described [79]. The full-length coding regions of *PRD3*, *ASY1*, *ASY3* and *PCH2* were PCR amplified from cDNA and cloned into Lv0 universal vector pICH41331 using primers listed in S12 Table. For fluorescent protein tagging, the Lv0 vectors with coding regions lacking stop codon were assembled in the Lv1 transient expression vector pICH47742, using the *35S* promoter vector (pICH51266), C-terminal tagging vectors (YFP and CFP), and *NOPALINE SYNTHASE GENE* (*NOS*) terminator vector (pICH41421). Plasmid DNA and protoplasts were prepared, as described [80]. To detect colocalization of CFP- and YFP-fusion proteins, 20 μg of total plasmid DNAs were co-transfected into approximately 20×10^3^ protoplasts and incubated at room temperature for 24 hours. As a negative control, *CFP-* or *YFP*-fusion plasmids alone were transfected. The fluorescence of transfected protoplasts was detected using a confocal microscope LSM 800, Zeiss.

### Cytological analysis

To visualize meiotic stages, inflorescences were fixed in 3:1 ethanol/glacial acetic acid at 4°C. Inflorescences were dissected in fixative solution then washed three times with citrate buffer (44.5 mM citric acid, 55.5 mM sodium citrate) for 2 minutes each. Buds were incubated in a digestion solution (0.33% (w/v) of cellulose, 0.33% (w/v) of pectolyase, citrate buffer) for 1hour and 30 minutes at 37°C. The enzyme solution was replaced by a solution of citrate buffer and each bud was gently moved to a microscope slide and mashed with a brass road. 10μl of 60% acetic acid was added and mixed with a needle. The slide was incubated on a hotplate at 48°C for 1 minute and 120μl of fixative solution was added. The slide was dried upside down with a hair dryer before adding a solution of DAPI and a cover glass. Fluorescence *in situ* hybridisation of *45S* and *5S* rDNA was carried out as previously described [81].

For BrdU pulse, stems of 6 week-old plants were cut under water and were submerged in a solution of 10 mM of BrdU (SIGMA). The stems were left for 2 hours under normal plant growth conditions to facilitate the incorporation of the BrdU into nuclear DNA via the transpiration streams. After 2 hours, the stems were transferred to water to start the pulse-chase experiment. Fixation of Inflorescences at defined time points and chromosome spread were performed as described above. Detection of BrdU was carried out with an α-BrdU antibody following the recommendations of the manufacturer (Roche).

Immunostaining of meiotic proteins was performed using fresh buds. Inflorescences were dissected on a damp filter paper under a stereo microscope and 6 buds at flower stage 9, as previously defined [82], were isolated and transferred to 5μl of enzyme digestion solution (0.4% cytohelicase, 1.5% sucrose, 1% polyvinylpyrollidone) on a microscopic slide. The buds were dissected to recover the anthers, while the rest of the bud tissue was discarded. The slide was then incubated in a moist box at 37°C for 1 minute and the anthers were gently open with a brass rod to release the meiocytes. 5μl of enzyme digestion solution was added, and the slide was incubated in a moist box at 37°C for 2 minutes. After this, 10μl of 1% lipsol was added and the solution was gently mixed with a needle for 1 minute before adding 20μl of 4% paraformaldehyde. The slides were then left to dry for 4 hours. Following this, the slides were washed for 5 minutes in a solution of PBST. 50μl of primary antibody (antibody diluted in 1% BSA in PBST) was added to the microscopic slides and the slides were incubated in a moist box at 4°C for 1 day. The slides were washed three times 5 minutes in PBST. A solution of secondary antibody (anti-fitc and anti-cy3 in 1% BSA in PBST) was added to the slides and the slides were incubated in a moist box at 37°C for 30 minutes. The slides were washed three times 5 minutes in PBST, air dried for 2 minutes and 10μl of DAPI solution was added. The following antibodies were used: α-ASY1 (rat/rabbit, 1/500 dilution) [24], α-ZYP1 (rabbit, 1/500 dilution) [50], α-RAD51 (rabbit, 1/500 dilution) [83], α-HA (rat, dilution 1/100) (Roche, 3F10), α-MYC (mouse, 1/50 dilution) (Santa Cruz Biotechnology, 9E10), α-REC8 (rabbit, 1/500 dilution) [28], α-ASY3 (rabbit, 1/500 dilution) [28], α-SMC3 (rat, 1/500 dilution) [28], α-γH2AX (Ser139, rabbit, 1/100 dilution) (Upstate Biotechnology, 07–164).

To count the recombination foci on the nuclei, the raw images were deconvolved using SoftWoRx software version 5.5 to increase the contrast and resolution of the signal. Deconvolved and raw images were put side by side and the foci were counted with ImageJ using the information from the two images. The degree of overlap localisation of PRD3-HA and SPO11-1-MYC with ASY1 was analyzed using SoftWoRx version 5.5 from Applied precision/GE Healthcare. Each cell was divided in cross-sections and the package “line profile” displayed a plot of fluorescence signal intensity for each cross-section. The fluorescence intensity for each recombination foci was then compared to the fluorescence of ASY1 axis. Overlapping fluorescence signals were assigned as co-localising foci/axis. For comparison with randomness, each image of PRD3-HA and SPO11-1-MYC was turned to 180-degree angle and the analysis was repeated.

Microscopy was conducted using a DeltaVision Personal DV microscope (Applied precision/GE Healthcare) equipped with a CDD Coolsnap HQ2 camera (Photometrics). Image capture was performed using SoftWoRx software version 5.5 (Applied precision/GE Healthcare). Image analysis and processing were performed using ImageJ.

### Yeast two-hybrid analysis

ASY1, ASY3, PCH2 and PRD3 CDS were cloned into pGADT7 and pGBKT7 and co-transformed to Y2HGold yeast cells using the Yeastmaker Yeast transformation System 2 (Clontech). Co-transformed yeast cells were selected on minimal medium lacking leucine and tryptophan (Clontech). To test for protein-protein interaction, yeast cells were transferred to low stringent selection (SD-LTH, lacking leucine, tryptophan and histidine; Clontech) or high stringent selection (SD-LTH, lacking leucine, tryptophan, histidine and adenine; Clontech) and incubated for 5 days at 30°C. To confirm the interaction, single colonies were grown overnight on minimal medium lacking leucine and tryptophan and the liquid cultures were used for serial drop dilutions where 3μl of undiluted, 10- and 100-fold diluted solution were spotted on each of the three selective medium and incubated at 30°C for 2 days.

### String association network

Brassica proteins identified from co-IP-MS were blast searched against Arabidopsis TAIR10 to identify the orthologue proteins. Arabidopsis proteins were then used as seeds to query the STRING database version 11.5 [56] and to establish a network of associating proteins. The interaction network was downloaded using the Cytoscape String Application [84] and clustering was done using the Cytoscape application named AutoAnnotate [85] based on the MCL clustering algorithm.

## Supporting information

S1 Fig γH2AX localisation in wild type prophase I. Localisation of ASY1 (green) with γH2AX (red) on wild type (Col) male meiocytes, from leptotene to pachytene stages. Scale bars = 10μM. Plot showing counts of γH2AX foci in wild type (Col) at the leptotene, zygotene and pachytene stages of meiosis. Black dots represent individual measurements, and red dots represent mean values.
(TIF)

S2 Fig PRD3-HA complements *prd3-3* phenotype. (**A**) Photograph of inflorescences from wild type (Col), *prd3-3* and *PRD3-HA prd3-3* plants. Scale bar = 5 cm. (**B**) Siliques from wild type (Col), *prd3-3* and *PRD3-HA prd3-3* plants. Scale bar = 2 cm. (**C**) DAPI staining of chromatin from wild type (Col), *prd3-3* and *PRD3-HA prd3-3* PMCs at the labelled stages. Scale bars = 10 μM. (**D**) α-HA western blotting of wild type and *PRD3-HA prd3-3* floral bud crude extracts, and Ponceau staining of the membrane showing equal loading of proteins between the samples. PRD3-HA has an expected molecular weight of 57 kDa.
(TIF)

S1 Table RAD51, γH2AX and SPO11-1-MYC foci count at leptotene, zygotene and pachytene. ASY1 and RAD51, ASY1 and γH2AX or ASY1 and SPO11-1-MYC were immunostained in Col. ASY1 staining was used to determine the meiotic stage and to count recombination foci on nuclei at a comparable stage.
(DOCX)

S2 Table SPO11-1-MYC foci count at leptotene in DSB defective mutant lines. ASY1 and SPO11-1-MYC were immunostained in DSB mutant lines. ASY1 staining was used to determine the meiotic stage and to count SPO11-1-MYC foci on nuclei at a comparable stage.
(DOCX)

S3 Table PRD3-HA foci count in Col and *asy3* at leptotene. PRD3-HA and ASY1 or SMC3 were immunostained in Col and *asy3*. ASY1 and SMC3 staining were used to determine the meiotic stage and to count PRD3-HA foci on nuclei at a comparable stage.
(DOCX)

S4 Table Co-localization analysis between ASY1 and PRD3-HA in wild-type or ASY1 and SPO11-1-MYC foci in wild type and *prd3* at leptotene. Co-localization between PRD3-HA and ASY1 in wild-type or ASY1 and SPO11-1-MYC foci in wild-type and *prd3* were quantified and the percentage of foci co-localizing or not co-localizing are reported. A Mann-Whitney-Wilcoxon test was performed to test for significance.
(DOCX)

S5 Table Co-localization analysis between ASY1 and randomised PRD3-HA or SPO11-1-MYC foci at leptotene. Image of PRD3-HA and SPO11-1-MYC were rotated 180 degrees to test for random overlap between PRD3-HA and ASY1 or SPO11-1-MYC and ASY1. Co-localization between random PRD3-HA and ASY1 or random SPO11-1-MYC foci and ASY1 axis were quantified and the frequency of foci co-localizing or not colocalizing are reported. A Mann-Whitney-Wilcoxon test was performed to test for significance.
(DOCX)

S6 Table List of proteins identified in the ASY1 co-immunoprecipitation experiment with a known role in meiosis. (DOCX)

S7 Table SPO11-1-MYC foci count in *spo11-1 SPO11-1-MYC* and *rec8 spo11-1 SPO11-1-MYC* in early prophase. ASY1 and SPO11-1-MYC were immunostained in *spo11-1 SPO11-1-MYC* and *rec8 spo11-1 SPO11-1-MYC* at letptotene stage. ASY1 staining was used to determine the meiotic stage and to count SPO11-1-MYC foci on cells at a comparable stage. A Mann-Whitney-Wilcoxon test was performed to test for significance.
(DOCX)

S8 Table γH2AX and RAD51 foci density per μm of axis in Col and *rec8*. ASY1 and γH2AX or RAD51 were co-stained on male meiosis in Col and *rec8*. γH2AX and RAD51 foci were counted and divided by the axis length (μm). A Mann-Whitney-Wilcoxon test was performed to test for significance.
(DOCX)

S9 Table γH2AX and RAD51 foci count in Col and *asy3/+* male meiosis at leptotene. ASY1 was immunostained with γH2AX or RAD51 in Col and *asy3-1/+* male meiosis at letptotene stage. ASY1 staining was used to determine the meiotic stage and to count γH2AX or RAD51 foci on nuclei at a comparable stage. A Mann-Whitney-Wilcoxon test was performed to test for significance.
(DOCX)

S10 Table Count of nuclei labelled or non-labelled with BrdU in Col and *prd3* time course experiment. Five time-points post BrdU-pulse were selected: T = 0h, T = 5h, T = 10h, T = 24h, T = 35h. Nuclei were counted for the following stages: interphase (Int), leptotene (Lep), zygotene/pachytene (Zyg/Pac), diplotene/metaphase I (Dip/MI), metaphase II/tetrad (MII/Tet). NL means "non-labelled” and L means “labelled”.
(DOCX)

S11 Table Amino acid length and [S/T]Q sites of proteins involved in DSB formation. (DOCX)

S12 Table Primers used to clone *ASY1*, *ASY3*, *PRD3* and *PCH2* into protoplast transient expression vectors. (DOCX)
