# Supplementary figures and images for: Differentiated function and localisation of SPO11-1 and PRD3 on the chromosome axis during meiotic DSB formation in Arabidopsis thaliana
Source: PLoS Genet. 2022 Jul 20;18(7):e1010298. doi: 10.1371/journal.pgen.1010298 (PMC9342770; doi:10.1371/journal.pgen.1010298)

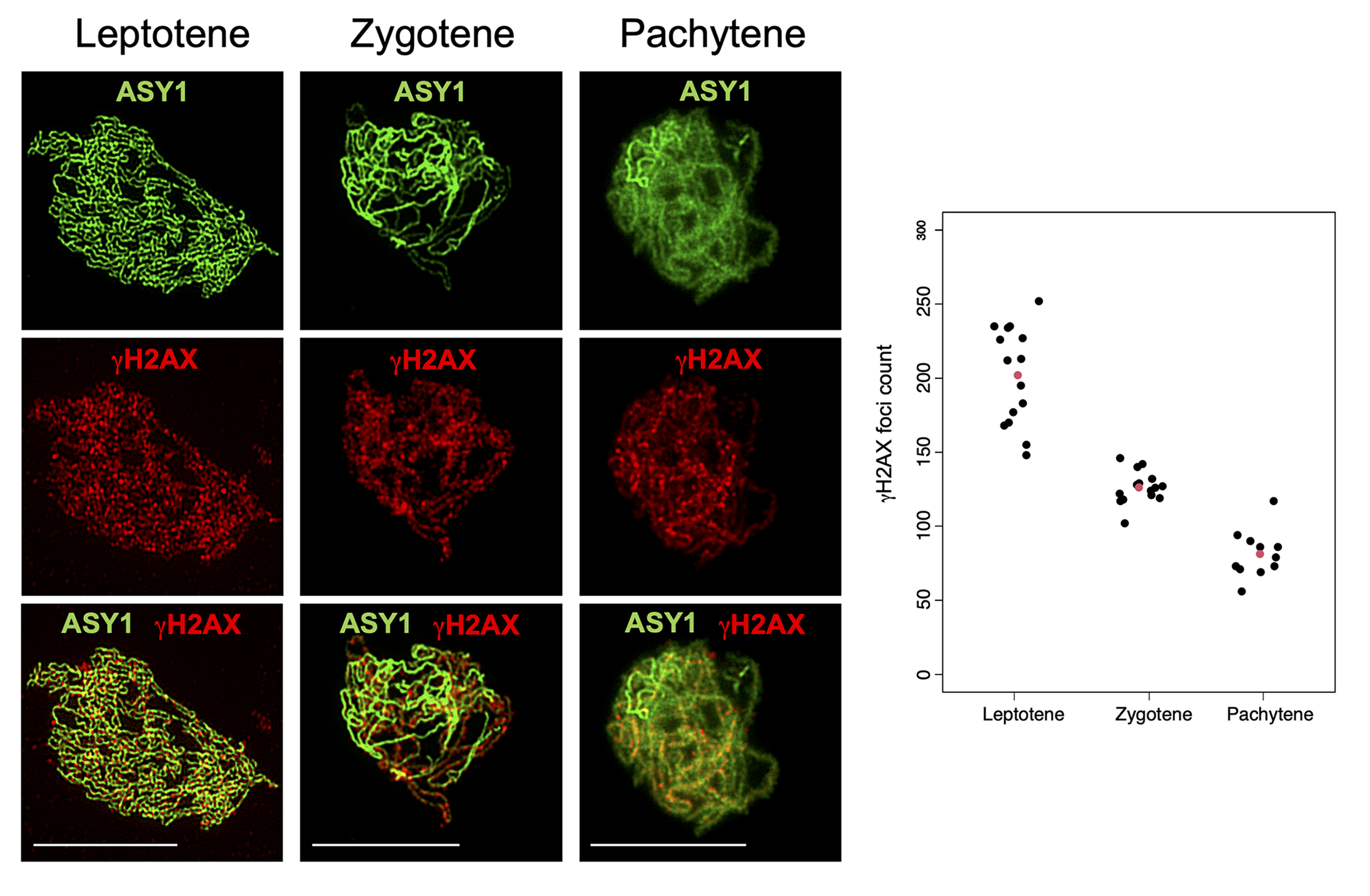

Supplement: S1 Fig — Localisation of ASY1 (green) with γH2AX (red) on wild type (Col) male meiocytes, from leptotene to pachytene stages. Scale bars = 10μM. Plot showing counts of γH2AX foci in wild type (Col) at the leptotene, zygotene and pachytene stages of meiosis. Black dots represent individual measurements, and red dots represent mean values. (TIF) [file pgen.1010298.s001.tif]

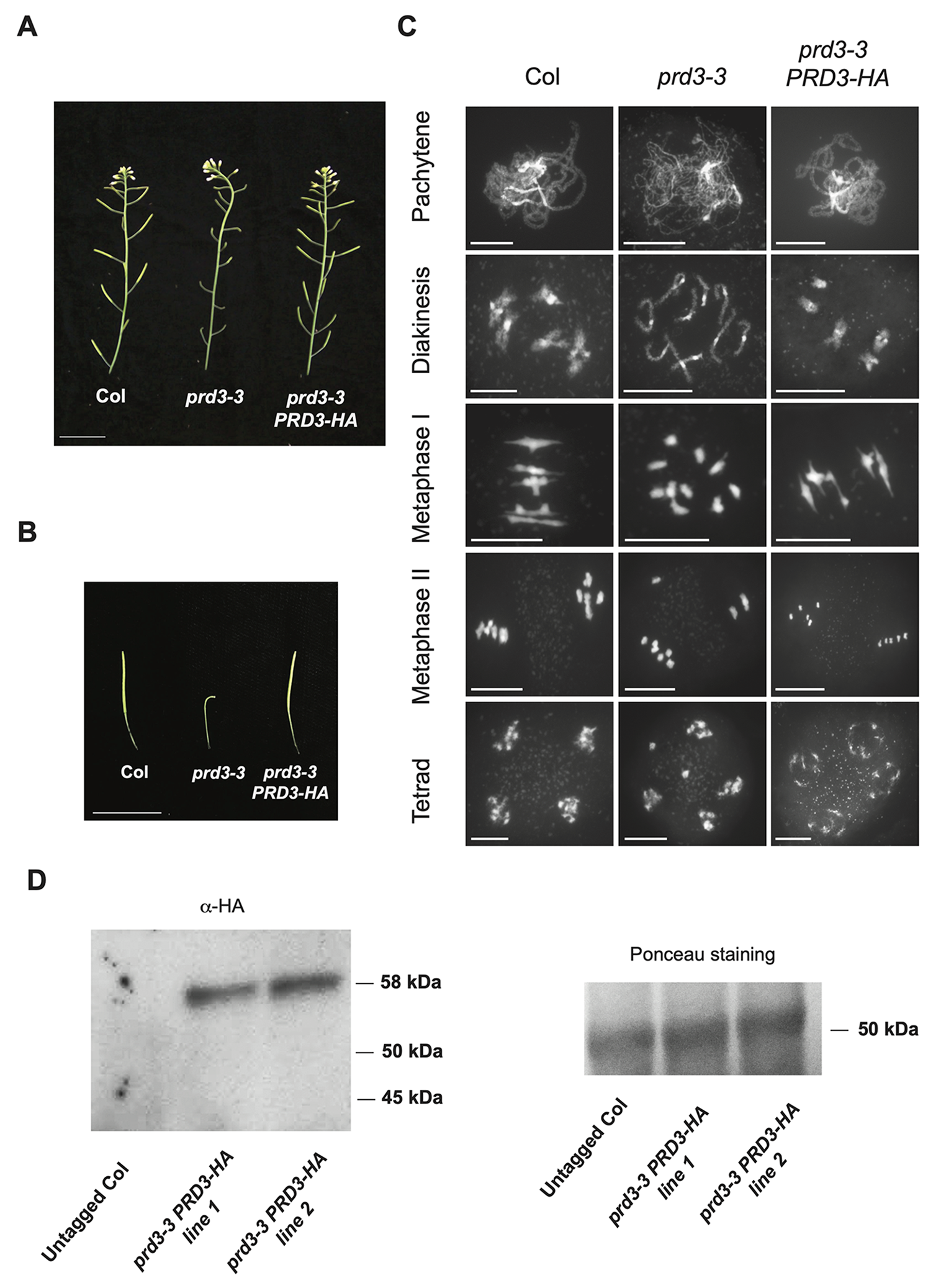

Supplement: S2 Fig — (A) Photograph of inflorescences from wild type (Col), prd3-3 and PRD3-HA prd3-3 plants. Scale bar = 5 cm. (B) Siliques from wild type (Col), prd3-3 and PRD3-HA prd3-3 plants. Scale bar = 2 cm. (C) DAPI staining of chromatin from wild type (Col), prd3-3 and PRD3-HA prd3-3 PMCs at the labelled stages. Scale bars = 10 μM. (D) α-HA western blotting of wild type and PRD3-HA prd3-3 floral bud crude extracts, and Ponceau staining of the membrane showing equal loading of proteins between the samples. PRD3-HA has an expected molecular weight of 57 kDa. (TIF) [file pgen.1010298.s002.tif]
