# Supplementary material for: Differentiated function and localisation of SPO11-1 and PRD3 on the chromosome axis during meiotic DSB formation in Arabidopsis thaliana
Source: PLoS Genet. 2022 Jul 20;18(7):e1010298. doi: 10.1371/journal.pgen.1010298 (PMC9342770; doi:10.1371/journal.pgen.1010298)
Supplement: S1 Table — ASY1 and RAD51, ASY1 and γH2AX or ASY1 and SPO11-1-MYC were immunostained in Col. ASY1 staining was used to determine the meiotic stage and to count recombination foci on nuclei at a comparable stage. (DOCX) [file pgen.1010298.s003.docx]

| Leptotene | | | Zygotene | | | Pachytene | | |
| --- | --- | --- | --- | --- | --- | --- | --- | --- |
| RAD51 | SPO11-1-MYC | γH2AX | RAD51 | SPO11-1-MYC | γH2AX | RAD51 | SPO11-1-MYC | γH2AX |
| 128 | 149 | 183 | 91 | 191 | 124 | 116 | 206 | 56 |
| 219 | 193 | 235 | 126 | 182 | 102 | 43 | 189 | 117 |
| 153 | 182 | 212 | 107 | 223 | 127 | 96 | 230 | 90 |
| 171 | 150 | 235 | 128 | 198 | 126 | 79 | 191 | 79 |
| 162 | 213 | 155 | 119 | 239 | 128 | 102 | 239 | 86 |
| 200 | 172 | 177 | 107 | 172 | 146 | 78 | 175 | 73 |
| 204 | 212 | 252 | 130 | 174 | 140 | 78 | 222 | 71 |
| 144 | 164 | 226 | 100 | 232 | 122 | 56 | 197 | 94 |
| 186 | 185 | 213 | 108 | 221 | 142 | 81 | 276 | 86 |
| 171 | 165 | 227 | 101 | 195 | 117 | 73 | 196 | 73 |
| 220 | 195 | 168 | 95 | 176 | 121 | 92 | 199 | 69 |
| 223 | 171 | 170 | 122 | 146 | 119 | 101 | 236 |  |
| 174 |  | 195 | 113 | 195 | 118 | 85 | 196 |  |
| 182 |  | 234 | 112 | 216 | 129 | 68 | 197 |  |
| 190 |  | 148 |  |  | 132 | 107 |  |  |
| 144 |  |  |  |  |  | 95 |  |  |
| 180 |  |  |  |  |  | 95 |  |  |
| 189 |  |  |  |  |  | 67 |  |  |
|  |  |  |  |  |  | 80 |  |  |
