# Supplementary material for: Differentiated function and localisation of SPO11-1 and PRD3 on the chromosome axis during meiotic DSB formation in Arabidopsis thaliana
Source: PLoS Genet. 2022 Jul 20;18(7):e1010298. doi: 10.1371/journal.pgen.1010298 (PMC9342770; doi:10.1371/journal.pgen.1010298)
Supplement: S2 Table — ASY1 and SPO11-1-MYC were immunostained in DSB mutant lines. ASY1 staining was used to determine the meiotic stage and to count SPO11-1-MYC foci on nuclei at a comparable stage. (DOCX) [file pgen.1010298.s004.docx]

| ***prd1*** | ***prd2*** | ***prd3*** | ***spo11-2*** | ***mtopVIb*** |
| --- | --- | --- | --- | --- |
| 159 | 140 | 250 | 147 | 74 |
| 161 | 115 | 217 | 132 | 94 |
| 158 | 166 | 201 | 103 | 93 |
| 235 | 116 | 128 | 88 | 72 |
| 175 | 135 | 192 | 92 | 40 |
| 146 | 175 | 187 | 102 | 44 |
| 181 | 163 | 102 | 102 | 62 |
| 167 | 134 | 214 | 93 | 54 |
| 217 | 126 | 143 | 77 | 87 |
| 165 | 174 | 213 | 81 | 60 |
| 140 | 121 | 195 | 104 | 50 |
| 169 | 127 | 180 | 106 | 47 |
| 147 | 138 | 174 | 88 | 59 |
|  | 116 | 171 | 82 | 66 |
|  |  | 174 | 86 | 69 |
