# Supplementary material for: Differentiated function and localisation of SPO11-1 and PRD3 on the chromosome axis during meiotic DSB formation in Arabidopsis thaliana
Source: PLoS Genet. 2022 Jul 20;18(7):e1010298. doi: 10.1371/journal.pgen.1010298 (PMC9342770; doi:10.1371/journal.pgen.1010298)
Supplement: S3 Table — PRD3-HA and ASY1 or SMC3 were immunostained in Col and asy3. ASY1 and SMC3 staining were used to determine the meiotic stage and to count PRD3-HA foci on nuclei at a comparable stage. (DOCX) [file pgen.1010298.s005.docx]

| **Col** | ***asy3*** |
| --- | --- |
| 129 | 81 |
| 155 | 73 |
| 156 | 62 |
| 155 | 77 |
| 160 | 87 |
| 152 | 110 |
| 131 | 89 |
| 166 | 60 |
| 174 | 76 |
| 131 | 87 |
|  | 57 |
|  | 75 |
|  | 63 |
|  | 71 |
|  | 69 |
