# Supplementary material for: Differentiated function and localisation of SPO11-1 and PRD3 on the chromosome axis during meiotic DSB formation in Arabidopsis thaliana
Source: PLoS Genet. 2022 Jul 20;18(7):e1010298. doi: 10.1371/journal.pgen.1010298 (PMC9342770; doi:10.1371/journal.pgen.1010298)
Supplement: S4 Table — Co-localization between PRD3-HA and ASY1 in wild-type or ASY1 and SPO11-1-MYC foci in wild-type and prd3 were quantified and the percentage of foci co-localizing or not co-localizing are reported. A Mann-Whitney-Wilcoxon test was performed to test for significance. (DOCX) [file pgen.1010298.s006.docx]

| **PRD3-HA and ASY1** | | **SPO11-1-MYC and ASY1** | | **SPO11-1-MYC and ASY1 in *prd3*** | |
| --- | --- | --- | --- | --- | --- |
| **Co-localize** | **Not co-localize** | **Co-localize** | **Not co-localize** | **Co-localize** | **Not co-localize** |
| 54.0 | 46.0 | 44.4 | 55.6 | 44.3 | 55.7 |
| 48.7 | 51.3 | 34.4 | 65.6 | 52.4 | 47.6 |
| 48.8 | 51.2 | 43.3 | 56.7 | 48.4 | 51.6 |
| 54.3 | 45.7 | 32.3 | 67.7 | 36.1 | 63.9 |
| 52.4 | 47.6 | 46.3 | 53.7 | 40.5 | 59.5 |
| 50.0 | 50.0 | 40.4 | 59.6 | 41.4 | 58.6 |
| 49.0 | 51.0 | 45.9 | 54.1 | 37.9 | 62.1 |
| 55.1 | 44.9 | 36.6 | 63.4 | 52.3 | 47.7 |
|  |  |  |  | 40.3 |  |
|  |  |  |  | 35.1 |  |
