# Supplementary material for: Differentiated function and localisation of SPO11-1 and PRD3 on the chromosome axis during meiotic DSB formation in Arabidopsis thaliana
Source: PLoS Genet. 2022 Jul 20;18(7):e1010298. doi: 10.1371/journal.pgen.1010298 (PMC9342770; doi:10.1371/journal.pgen.1010298)
Supplement: S5 Table — Image of PRD3-HA and SPO11-1-MYC were rotated 180 degrees to test for random overlap between PRD3-HA and ASY1 or SPO11-1-MYC and ASY1. Co-localization between random PRD3-HA and ASY1 or random SPO11-1-MYC foci and ASY1 axis were quantified and the frequency of foci co-localizing or not colocalizing are reported. A Mann-Whitney-Wilcoxon test was performed to test for significance. (DOCX) [file pgen.1010298.s007.docx]

| **Random PRD3-HA and ASY1** | | **Random SPO11-1-MYC and ASY1** | |
| --- | --- | --- | --- |
| **Co-localize** | **Not co-localize** | **Co-localize** | **Not co-localize** |
| 21.2 | 78.8 | 28.9 | 71.1 |
| 25.8 | 74.2 | 25.7 | 74.3 |
| 24.5 | 75.5 | 28.2 | 71.8 |
| 20.6 | 79.4 | 33.3 | 66.7 |
| 22.0 | 78.0 | 21.4 | 78.57 |
| 20.6 | 79.4 | 19.9 | 80.14 |
| 27.2 | 72.8 | 13.4 | 86.60 |
| 24.8 | 75.2 | 27.0 | 73.05 |
