# Supplementary material for: Differentiated function and localisation of SPO11-1 and PRD3 on the chromosome axis during meiotic DSB formation in Arabidopsis thaliana
Source: PLoS Genet. 2022 Jul 20;18(7):e1010298. doi: 10.1371/journal.pgen.1010298 (PMC9342770; doi:10.1371/journal.pgen.1010298)
Supplement: S6 Table — (DOCX) [file pgen.1010298.s008.docx]

| ASY1 |
| --- |
| PCH2 |
| PDS5C |
| SMC1 |
| ZYP1a |
| SMC3 |
| SCC3 |
| MCM2 |
| CAP-D2 |
| ICU2 |
| ASY4 |
| PRD3 |
| TOPII |
| DMC1 |
| CDKA;1 |
| ASY3 |
| AGO4 |
