# Supplementary material for: Differentiated function and localisation of SPO11-1 and PRD3 on the chromosome axis during meiotic DSB formation in Arabidopsis thaliana
Source: PLoS Genet. 2022 Jul 20;18(7):e1010298. doi: 10.1371/journal.pgen.1010298 (PMC9342770; doi:10.1371/journal.pgen.1010298)
Supplement: S7 Table — ASY1 and SPO11-1-MYC were immunostained in spo11-1 SPO11-1-MYC and rec8 spo11-1 SPO11-1-MYC at letptotene stage. ASY1 staining was used to determine the meiotic stage and to count SPO11-1-MYC foci on cells at a comparable stage. A Mann-Whitney-Wilcoxon test was performed to test for significance. (DOCX) [file pgen.1010298.s009.docx]

| **Col** | ***rec8*** |
| --- | --- |
| 254 | 233 |
| 193 | 159 |
| 264 | 200 |
| 182 | 177 |
| 150 | 136 |
| 213 | 171 |
| 172 | 213 |
| 216 | 164 |
| 212 | 210 |
| 164 | 187 |
| 185 | 153 |
| 267 | 188 |
| 195 |  |
