# Supplementary material for: Differentiated function and localisation of SPO11-1 and PRD3 on the chromosome axis during meiotic DSB formation in Arabidopsis thaliana
Source: PLoS Genet. 2022 Jul 20;18(7):e1010298. doi: 10.1371/journal.pgen.1010298 (PMC9342770; doi:10.1371/journal.pgen.1010298)
Supplement: S8 Table — ASY1 and γH2AX or RAD51 were co-stained on male meiosis in Col and rec8. γH2AX and RAD51 foci were counted and divided by the axis length (μm). A Mann-Whitney-Wilcoxon test was performed to test for significance. (DOCX) [file pgen.1010298.s010.docx]

| **γH2AX foci density per μm of axis** | | **RAD51 foci density per μm of axis** | |
| --- | --- | --- | --- |
| **Col** | ***rec8*** | **Col** | ***rec8*** |
| 0.84 | 1.16 | 0.75 | 1.77 |
| 1.00 | 1.47 | 0.78 | 1.67 |
| 0.90 | 1.20 | 0.74 | 1.72 |
| 0.99 | 1.86 | 0.79 | 2.10 |
| 0.86 | 2.88 | 0.74 | 2.06 |
| 0.99 | 1.73 | 0.99 | 1.29 |
| 1.12 | 1.90 | 0.76 | 1.36 |
| 0.99 | 1.82 | 0.82 | 1.55 |
| 0.98 | 1.40 | 0.89 | 1.45 |
| 0.88 | 3.98 | 1.03 | 1.61 |
| 0.86 | 1.85 | 0.97 | 1.75 |
| 0.78 | 1.45 | 0.73 | 0.88 |
| 0.92 | 1.27 | 0.87 | 1.16 |
| 1.01 | 1.51 | 0.75 | 1.42 |
| 0.92 | 1.07 | 0.70 | 1.56 |
