# Supplementary material for: Differentiated function and localisation of SPO11-1 and PRD3 on the chromosome axis during meiotic DSB formation in Arabidopsis thaliana
Source: PLoS Genet. 2022 Jul 20;18(7):e1010298. doi: 10.1371/journal.pgen.1010298 (PMC9342770; doi:10.1371/journal.pgen.1010298)
Supplement: S9 Table — ASY1 was immunostained with γH2AX or RAD51 in Col and asy3-1/+ male meiosis at letptotene stage. ASY1 staining was used to determine the meiotic stage and to count γH2AX or RAD51 foci on nuclei at a comparable stage. A Mann-Whitney-Wilcoxon test was performed to test for significance. (DOCX) [file pgen.1010298.s011.docx]

| **γH2AX** | | **RAD51** | |
| --- | --- | --- | --- |
| **Col** | ***asy3-1/+*** | **Col** | ***asy3-1/+*** |
| 183 | 96 | 153 | 98 |
| 235 | 129 | 134 | 144 |
| 212 | 122 | 175 | 104 |
| 235 | 135 | 142 | 127 |
| 155 | 147 | 131 | 125 |
| 177 | 123 | 128 | 127 |
| 252 | 101 | 147 | 128 |
| 226 | 113 | 160 | 141 |
| 213 | 91 | 225 | 113 |
| 227 | 76 | 166 | 125 |
| 168 | 89 | 175 | 143 |
| 170 | 113 | 192 | 126 |
| 195 |  |  | 122 |
| 234 |  |  | 120 |
| 148 |  |  | 141 |
