# Supplementary material for: Differentiated function and localisation of SPO11-1 and PRD3 on the chromosome axis during meiotic DSB formation in Arabidopsis thaliana
Source: PLoS Genet. 2022 Jul 20;18(7):e1010298. doi: 10.1371/journal.pgen.1010298 (PMC9342770; doi:10.1371/journal.pgen.1010298)
Supplement: S10 Table — Five time-points post BrdU-pulse were selected: T = 0h, T = 5h, T = 10h, T = 24h, T = 35h. Nuclei were counted for the following stages: interphase (Int), leptotene (Lep), zygotene/pachytene (Zyg/Pac), diplotene/metaphase I (Dip/MI), metaphase II/tetrad (MII/Tet). NL means "non-labelled” and L means “labelled”. (DOCX) [file pgen.1010298.s012.docx]

|  | Int NL | Int L | Lep NL | Lep L | Zyg/Pac NL | Zyg/Pac L | Dip/MI NL | Dip/MI L | MII/Tet NL | MII/Tet L |
| --- | --- | --- | --- | --- | --- | --- | --- | --- | --- | --- |
| Wild-type T=0 | 17 | 27 (61%) | 10 | 0 (0%) | 13 | 0 (0%) | ND | ND | ND | ND |
| Wild-type T=5 | 16 | 34 (68%) | 5 | 4 (44%) | 0 | 31 (0%) | ND | ND | ND | ND |
| Wild-type T=10 | 1 | 17 (95%) | 1 | 9 (90%) | 55 | 4 (7%) | ND | ND | ND | ND |
| Wild-type T=24 | ND | ND | 0 | 15 (100%) | 6 | 50 (89%) | 14 | 0 (0%) | ND | ND |
| Wild-type T=35 | ND | ND | ND | ND | ND | ND | ND | ND | 8 | 12 (60%) |
| Atprd3  T=0 | 19 | 24 (56%) | 10 | 0 (0%) | 16 (100%) | 0 | ND | ND | ND | ND |
| Atprd3  T=5 | 15 | 40 (73%) | 7 | 8 (53%) | 69 | 0 (0%) | ND | ND | ND | ND |
| Atprd3  T=10 | 3 | 24 (89%) | 0 | 10 (100%) | 53 | 2 (4%) | ND | ND | ND | ND |
| Atprd3  T=24 | ND | ND | 0 | 14 (100%) | 21 | 62 (75%) | 18 | 0 (0%) | ND | ND |
| Atprd3  T=35 | ND | ND | ND | ND | ND | ND | ND | ND | 14 | 6 (30%) |
