# Supplementary material for: Differentiated function and localisation of SPO11-1 and PRD3 on the chromosome axis during meiotic DSB formation in Arabidopsis thaliana
Source: PLoS Genet. 2022 Jul 20;18(7):e1010298. doi: 10.1371/journal.pgen.1010298 (PMC9342770; doi:10.1371/journal.pgen.1010298)
Supplement: S11 Table — (DOCX) [file pgen.1010298.s013.docx]

| **Protein** | **[S/T]Q sites / protein length** |
| --- | --- |
| SPO11-1 | 1/362 |
| SPO11-2 | 1/383 |
| MTOPVIB | 5/493 |
| PRD1 | 10/1268 |
| PRD2 | 3/385 |
| PRD3 | 10/449 |
| DFO | 0/233 |
