# Supplementary material for: Differentiated function and localisation of SPO11-1 and PRD3 on the chromosome axis during meiotic DSB formation in Arabidopsis thaliana
Source: PLoS Genet. 2022 Jul 20;18(7):e1010298. doi: 10.1371/journal.pgen.1010298 (PMC9342770; doi:10.1371/journal.pgen.1010298)
Supplement: S12 Table — (DOCX) [file pgen.1010298.s014.docx]

| **oligo name** | **sequence** |
| --- | --- |
| PRD3 Lv0 F(AATG) | CCGAAGACGGCTCAAATGATGAAGATGAATATTAACAAAGCCTGCGATCTG |
| PRD3 overlap F1 | CCCCAAAGATACAAGTGGGATGTTGGAAAACGGTGAAACCAGAAAAAAGCAATTTCAAG |
| PRD3 overlap R1 | CTTGAAATTGCTTTTTTCTGGTTTCACCGTTTTCCAACATCCCACTTGTATCTTTGGGG |
| PRD3 Lv0 R(ggTTCG) | CCGAAGACGGCTCGCGAACCGTTAATTATTATGGGGTTACCGAACTTCCTCTTTG |
| ASY1 Lv0 F(AATG) | CCGAAGACGGCTCAAATGATGGTGATGGCTCAGAAGCTG |
| ASY1 overlap F1 | AGGAAATATTTGAAaACGCTCATGTTCAG |
| ASY1 overlap R1 | CTGAACATGAGCGTtTTCAAATATTTCCT |
| ASY1 overlap F2 | AGAGGCTGTACAGAgGACGAAGCTCAGTA |
| ASY1 overlap R2 | TACTGAGCTTCGTCcTCTGTACAGCCTCT |
| ASY1 overlap F3 | TCTGTTGCTCCTGAgGACTACTTGTACAT |
| ASY1 overlap R3 | ATGTACAAGTAGTCcTCAGGAGCAACAGA |
| ASY1 Lv0 R(ggTTCG) | ccGAAGACggCTCGCGAAccATTAGCTTGAGATTTCTGACG |
| ASY3 Lv0 F(AATG) | CCGAAGACGGCTCAAATGAGCGACTATAGAAGCTTCGG |
| ASY3 overlap F1 | GACCTCACCTTGGAGGTCACCGAGATCGTCTC |
| ASY3 overlap R1 | GAGACGATCTCGGTGACCTCCAAGGTGAGGTC |
| ASY3 overlap F2 | GTGAACTCTGAAACCCCAGAAGTTGAAAAGACCAACTTCAAG |
| ASY3 overlap R2 | GAAGTTGGTCTTTTCAACTTCTGGGGTTTCAGAGTTCACATC |
| ASY3 Lv0 R(ggTTCG) | CCGAAGACGGCTCGCGAACCATCATCCCTCAAACATTCTGCGAC |
| PCH2 Lv0 F(AATG) | CCGAAGACGGCTCAAATGGTGGAGGACCCGATTCCTCTTCCAAACGCTTCCATGGAAGTC |
| PCH2 Lv0 R(ggTTCG) | CCGAAGACGGCTCGCGAACCTTCAGGTTGTTCAGACTTCTCTC |
